# Arkadia/RNF111 is a SUMO-targeted ubiquitin ligase with preference for substrates marked with SUMO1-capped SUMO2/3 chain

**DOI:** 10.1038/s41467-019-11549-3

**Published:** 2019-08-15

**Authors:** Annie M. Sriramachandran, Katrin Meyer-Teschendorf, Stefan Pabst, Helle D. Ulrich, Niels H. Gehring, Kay Hofmann, Gerrit J. K. Praefcke, R. Jürgen Dohmen

**Affiliations:** 1 Institute for Genetics, Faculty of Mathematics and Natural Sciences, University of Cologne, Center of Molecular Biosciences, Zülpicher Str. 47a, D-50674 Cologne, Germany; 2 Institute of Molecular Biology, Ackermannweg 4, D-55128 Mainz, Germany; 3 Center for Molecular Medicine Cologne (CMMC), University of Cologne, Robert-Koch-Straße 21, D-50931 Cologne, Germany; 4 Present Address: Paul-Ehrlich-Institute, Department of Haematology and Transfusion Medicine, Paul-Ehrlich-Str. 51-59, D-63225 Langen, Germany

**Keywords:** Ubiquitylation, Ligases, PML bodies, Sumoylation

## Abstract

Modification with SUMO regulates many eukaryotic proteins. Down-regulation of sumoylated forms of proteins involves either their desumoylation, and hence recycling of the unmodified form, or their proteolytic targeting by ubiquitin ligases that recognize their SUMO modification (termed STUbL or ULS). STUbL enzymes such as Uls1 and Slx5-Slx8 in budding yeast or RNF4 and Arkadia/RNF111 in humans bear multiple SUMO interaction motifs to recognize substrates carrying poly-SUMO chains. Using yeast as experimental system and isothermal titration calorimetry, we here show that Arkadia specifically selects substrates carrying SUMO1-capped SUMO2/3 hybrid conjugates and targets them for proteasomal degradation. Our data suggest that a SUMO1-specific binding site in Arkadia with sequence similarity to a SUMO1-binding site in DPP9 is required for targeting endogenous hybrid SUMO conjugates and PML nuclear bodies in human cells. We thus characterize Arkadia as a STUbL with a preference for substrate proteins marked with distinct hybrid SUMO chains.

## Abstract (web summary)

The cellular functions of poly-SUMO chains of different compositions are not fully understood. Here, the authors characterize Arkadia/RNF111 as a SUMO-targeted ubiquitin ligase that recognizes proteins with hybrid SUMO1-capped SUMO2/3 chains and targets them for proteasomal degradation.

## Introduction

Posttranslational modification of proteins by conjugation of the ubiquitin-related modifier SUMO to other proteins (termed sumoylation) is essential for viability in most eukaryotes and has many important functions^1–3^. Sumoylation of proteins can alter their interaction properties and thereby their subcellular localization, function, and/or stability^4^. We and others have identified a class of RING-type ubiquitin ligases, named SUMO-targeted ubiquitin ligases (STUbLs), that recognize and ubiquitylate SUMO modified proteins^5–11^. Two such STUbLs, Uls1 and the heterodimeric Slx5-Slx8 (Uls2), which bear multiple short SUMO interaction motifs (SIMs), were identified in *Saccharomyces cerevisiae*^5,6^. They mediate the degradation of poly-sumoylated proteins by the proteasome^5^. In a similar mechanism, the human RING finger protein RNF4 was shown to mediate arsenic-induced and SUMO-dependent degradation of the promyelocytic leukemia (PML) protein or of its oncogenic variant PML-RARα. The latter occurs in acute promyelocytic leukemia as the consequence of a chromosomal translocation^9,10,12^. RNF4, via its at least three SIMs, was shown to preferentially bind to SUMO chains, with a slight preference for chains made of SUMO2^9,13–15^. Previous work has revealed that, similar to *S. cerevisiae* Smt3 (the only SUMO type present in this yeast), the SUMO paralogs SUMO2 and SUMO3 (which are nearly identical and therefore hereafter referred to as SUMO2/3) form chains efficiently in vivo and in vitro^16,17^. Chain formation mainly involves formation of isopeptide bonds between the C-terminal Gly residue of one SUMO2/3 molecule and the Lys-11 (K11) residue of another. K11 is part of a consensus site for efficient sumoylation (I/L/V-K-x-D/E)^1,17^. SUMO1 lacks such a Lys residue in a consensus site and hence does not form chains efficiently^17^. Instead, based upon proteomic data, it was suggested that SUMO1 could serve as a chain terminator upon its attachment to K11 of the distal SUMO2/3 moiety^18^. Poly-SUMO2/3 conjugates become particularly prominent in cells subjected to stress conditions such as treatment with heat, hydroxyurea, arsenic trioxide, H_2_O_2_, or ethanol suggesting that it is one function of SUMO chains to eliminate certain proteins in STUbL-dependent mechanisms^12,19–22^. Conditions that may favor the generation of SUMO1-capped SUMO2/3 chains have not been identified up to this point. Our discovery described below, however, indicates that such capped chains confer a distinct targeting signal for ubiquitylation.

In the present study, we report our characterization of the human RING finger protein Arkadia (also known as RNF111), a protein that is known to promote TGF-β (transforming growth factor β) signaling^23,24^, as a STUbL with a distinct, previously unknown specificity for proteins carrying mixed SUMO chains. We use isothermal titration calorimetry (ITC) and the yeast *S. cerevisiae* to investigate Arkadia’s substrate specificity in vitro and in vivo. Using engineered test substrates bearing different types of poly-SUMO chains, we discover that Arkadia represents a distinct type of STUbL with a preference for substrates carrying hybrid SUMO1-capped SUMO2/3 chains.

## Results

### Bioinformatical characterization of Arkadia as a human STUbL

**Fig. 1:**
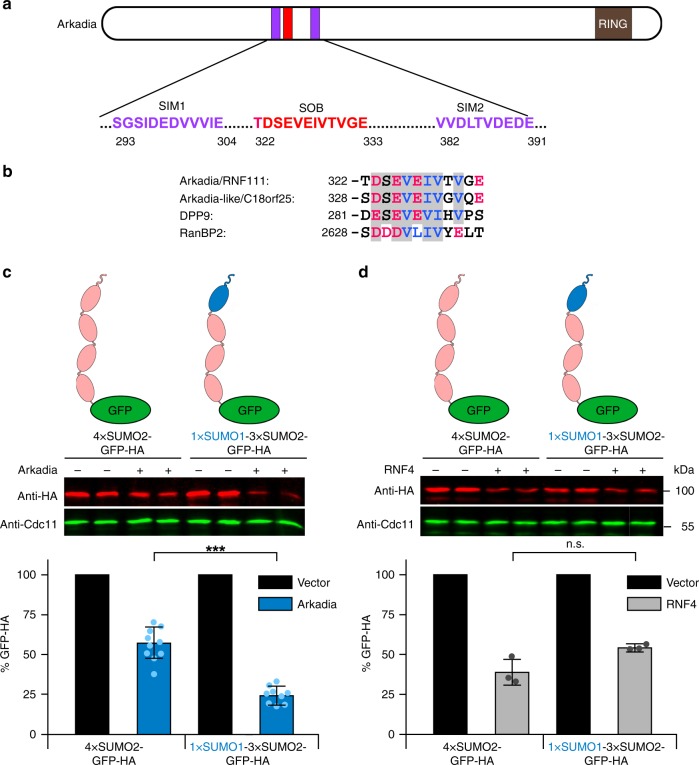
Arkadia is a STUbL with a substrate preference distinct from RNF4. **a** Schematic representation of relevant domains and their positions in human Arkadia. **b** Alignment of SUMO-binding motifs from Arkadia, Arkadia-like 1 (ARKL1), DPP9, and RanBP2. Residues that are similar in at least the first three of the molecules are shaded in gray. Acidic residues are shown in red, hydrophobic residues are shown in blue. **c** Analysis of substrate targeting by Arkadia upon expression in *S. cerevisiae*. HA-tagged GFP test substrates fused with their N-terminal ends to either 4xSUMO2 or a SUMO1-capped 3xSUMO2 chain were expressed in cells either transformed with an empty vector or with a plasmid expressing Arkadia. Cells were grown to log phase and extract proteins analyzed by quantitative anti-HA Western blotting. Endogenous Cdc11 protein levels were detected for normalization of loaded protein amounts. The middle panels show the Western blot images obtained for two independent transformants of each plasmid combination. The bottom panels show the results of quantifications obtained with data for nine independent transformants each. Bars represent the means. Standard deviations (SD) are indicated by error bars. **d** Same as in **c**, but for RNF4 obtained with three independent transformants each. Three asterisks indicate significant differences with *p*-values < 0.001. n.s., not significant. Statistical significance was determined with unpaired and two-sided Student’s *t*-tests. Source data are provided as a Source Data file

Using a previously described approach^25^, we screened the databases for human proteins that bear RING finger motifs characterizing them as ubiquitin ligases, and SIMs that suggest an interaction with sumoylated proteins. One of the proteins identified was Arkadia (also known as RING finger protein 111, RNF111), a ubiquitin ligase that enhances TGF-β signaling by promoting the degradation of repressors of target gene expression such as SnoN and Ski^23^. As was also noted by others^26–28^, Arkadia bears multiple SUMO interaction motifs (Fig. 1a). A motif encompassing residues 293–304 (SIM1) represents a typical reversed (type r) SIM, and another motif (residues 382–391; SIM2) represents a typical type b SIM^25^. An additional motif (residues 322–333) combines features of a reversed (type r) SIM with those of a SUMO1-specific interaction motif that has been discovered in the human DPP9 protein (Fig. 1b)^29^. For reasons described below, we refer to this sequence of Arkadia as SUMO one binding (SOB) motif.

### Preference of Arkadia for SUMO1-capped SUMO2 chains

Similar to what has been described for STUbLs in budding and fission yeasts as well as for RNF4 in human cells, the presence of both a RING domain and multiple SUMO-binding motifs suggested that Arkadia could have a similar selectivity toward poly-sumoylated proteins. To test this possibility, we employed a heterologous in vivo assay, in which *S. cerevisiae* cells expressing reporter constructs encoding HA epitope-tagged GFP substrates as translational fusions with uncleavable SUMO chains are used to monitor the activity of the human STUbL. Initially, we used a reporter protein bearing GFP N-terminally tagged with a chain of four SUMO2 moieties (FLAG-4xSUMO2-GFP-HA)^13^. The N-terminal SUMO2 moiety of this test substrate carried the authentic N-terminus extended by a FLAG tag, whereas the internal SUMO2 units lacked the N-terminal 11 amino acids^9,13^. Studies on the properties of RNF4 had shown that such a linear SUMO2 chain is recognized with a similar efficiency as a native lysine-linked SUMO2 chain^9,14^. Another study revealed that SUMO1 can be found attached to SUMO2 in human cells probably acting as an inhibitor of chain elongation^18^. We therefore generated a second reporter bearing a linear SUMO1-capped SUMO2 chain (FLAG-SUMO1-3xSUMO2-GFP-HA). Plasmids encoding these reporter proteins were introduced into *S. cerevisiae* cells together with an empty vector or plasmids expressing either Arkadia or RNF4. In comparison to the vector control, co-expression of Arkadia led to a small but significant reduction (to ~60%) of steady-state levels of the 4xSUMO2-GFP reporter (Fig. 1c). Co-expression of RNF4 led to a slightly stronger reduction of this reporter protein (to ~40%) compared to the vector control (Fig. 1d). For the 1xSUMO1-3xSUMO2-GFP reporter protein, we surprisingly observed that Arkadia caused a much stronger reduction (to ~25%) of the steady-state levels (Fig. 1c), whereas the effect of RNF4 on this reporter was less pronounced than its effect on the 4xSUMO2-GFP protein (Fig. 1d). These different effects were apparently not due to differences in the subcellular localization, as both types of substrates and both STUbLs displayed a similar distribution between cytosol and nucleus (Supplementary Fig. 1). Together, these data indicated that, in contrast to RNF4, Arkadia has a strong preference for substrates bearing a SUMO1-capped SUMO2 chain. Pulse chase protein stability assays with cells expressing 1xSUMO1-3xSUMO2-GFP verified that Arkadia enhances the degradation of this reporter protein, which however is already an unstable protein in the absence of Arkadia (Supplementary Fig. 2a). The latter is apparently due to the activity of the endogenous yeast STUbLs as the turnover was inhibited in *slx5 uls1* double mutants (Supplementary Fig. 2b). Degradation of the test substrate, both in the absence and presence of Arkadia, was strongly inhibited by the proteasome inhibitor MG132 (Supplementary Fig. 2a). The notion that Arkadia promotes degradation of the GFP substrate carrying a SUMO1-capped SUMO2 chain by mediating its ubiquitylation was further corroborated by pulldown experiments that revealed a strong increase of ubiquitin-modified forms of the substrate upon co-expression of Arkadia (Supplementary Fig. 2c).

### Arkadia preferentially binds oligomeric SUMO1-capped chains

**Fig. 2:**
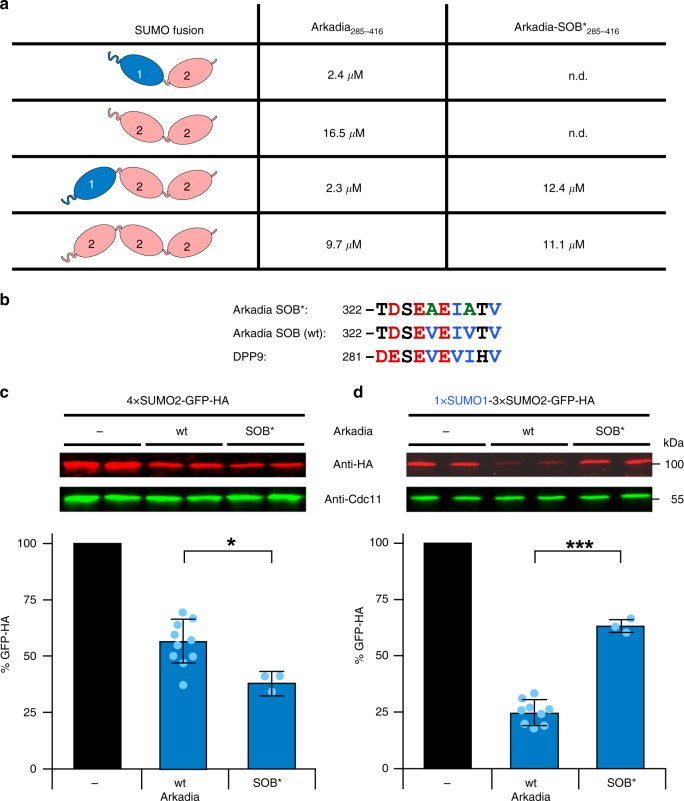
A SUMO1-specific binding site (SOB) in Arkadia mediates specificity. **a** Dissociation constants (*K*_D_) for the dimeric and trimeric SUMO chains listed derived from ITC measurements such as the ones shown in Supplementary Fig. 3. N.d., not determined. **b** Depiction of the wild-type SOB sequence and its mutant version (SOB*), in which two Val residues were mutated to Ala in an alignment with a similar motif in DPP9. **c**, **d**, Comparison of targeting efficiencies of the same substrates as in Fig. 1c by wild-type and SOB* versions of Arkadia upon their co-expression in *S. cerevisiae*. The analysis was carried out as described in Fig. 1c. Bars represent the means. Standard deviations (SD) are indicated by error bars. Quantifications are derived from experiments with nine independent transformants for wild-type Arkadia (same as in Fig. 1c) and three independent transformants for its SOB* mutant version. Asterisks indicate significant differences with *p*-values < 0.02 (one), *p*-values < 0.001 (three). Statistical significance was determined with unpaired and two-sided Student’s *t*-tests. Source data for **a**, **c**, and **d** are provided as a Source Data file

In order to further characterize the substrate preferences of Arkadia, we determined the affinity of linear SUMO dimers or trimers to the SUMO-binding domain of Arkadia by ITC (Fig. 2a and Supplementary Fig. 3). SUMO2 dimers and trimers bound with an affinity (dissociation constant *K*_D_ 10–17 µM, Fig. 2a) similar to values determined by surface plasmon resonance^28^ and to the values we have previously determined for RNF4^12,13^. SUMO1-capped dimers or trimers, in comparison, both bound at least fourfold more strongly (*K*_D_ 2–3 µM). These data therefore indicated that the preference for substrates marked with SUMO1-capped SUMO2 chains observed in the in vivo assays described above are at least in part determined within the SUMO-binding domain (residues 285–416) of Arkadia. The results further demonstrated that this domain in isolation is sufficient to recognize a SUMO dimer with no further enhancement of binding affinity by an extension of the chain to a trimer.

### Arkadia exhibits a preference for longer SUMO chains

To investigate the SUMO chain length requirements of Arkadia in a cellular context, we generated GFP-based test substrates with one to three SUMO moieties. A single SUMO1 moiety did not cause a detectable Arkadia-mediated targeting of a GFP-HA reporter (Supplementary Fig. 4a). A dimeric SUMO1–SUMO2 hybrid chain, in contrast, already caused a significant reduction of the reporter (to 68%) by Arkadia as compared to the vector control (Supplementary Fig. 4b). The levels of a substrate bearing a trimeric SUMO1-capped SUMO2 chain were further reduced to ~49% by co-expressed Arkadia (Supplementary Fig. 4b) indicating that a trimeric chain is more efficient than a dimeric one but less efficient than a tetrameric chain in mediating targeting by Arkadia, the latter of which caused reduction to ~25% of the reporter (Fig. 1c). In comparison, Arkadia-mediated targeting of substrates bearing homogeneous SUMO2 chains was much less efficient, with no effect of dimeric chains, reduction to ~78% by trimeric chains and to ~56% by tetrameric chains (Fig. 1c and Supplementary Fig. 4b). In summary, Arkadia has a preference for substrates tagged with SUMO1-capped chains, and the targeting efficiency increases with chain length. The observation that a trimeric SUMO1-capped SUMO2 chain yielded a significantly better targeting than a SUMO1–SUMO2 dimeric chain in vivo (Supplementary Fig. 4b) was in contrast to the observed identical affinity of the Arkadia SUMO-binding domain (residues 285–416) to such chains in vitro (Fig. 2). These findings point either to a contribution of other parts of Arkadia or to a cooperation of the two SUMO-binding domains of an Arkadia dimer in the detection of a larger number of SUMO moieties resulting in a preference for substrates tagged with longer chains (see “Discussion”).

The results described above raised additional questions. One of them was whether SUMO1 chains, even though they are expected to form less efficiently in cells (see “Introduction”), would also lead to targeting by Arkadia. Another one was whether presence of SUMO1 at the distal end of a chain is of importance. To address these issues, we generated and tested two additional substrates. The first one contains a chain of four SUMO1 moieties (4xSUMO1-GFP-HA), the second one has a SUMO2-capped 3xSUMO1 chain. Both types of substrates were relatively poorly targeted by Arkadia upon co-expression in yeast (Supplementary Fig. 5). These results indicate that, while SUMO1 is important at the distal end of a SUMO2 chain for efficient targeting by Arkadia, SUMO1 chains do not represent good targeting signals for this STUbL supporting our notion that SUMO1-capped SUMO2 chains are the preferred recognition signal of this ligase.

The preference for substrates carrying multiple SUMO moieties, in addition, raised the question whether they necessarily need to be presented in form of a chain (polysumoylation), or whether the presence of multiple single moieties on the same protein (multi-[mono]-sumoylation) would be sufficient for recognition by Arkadia. To address this question, we designed a test protein with defined multiple SUMO-attachments that could be tested in our experimental system. To achieve this, we introduced a domain from cartilage oligomeric matrix protein (COMP), which forms a five-stranded coiled coil^30^, into various of our GFP substrates upstream of the C-terminal HA tag. As a result, pentameric SUMO1-GFP and SUMO2-GFP substrates are formed that present multiple SUMO moieties. The oligomeric state of these substrates was verified by native PAGE (Supplementary Fig. 6a). As a control, we generated a SUMO1-3xSUMO2-GFP-COMP-HA to ascertain that introduction of the 43 amino acid oligomerization domain did not interfere with targeting by Arkadia. As expected, Arkadia targeted the latter protein, whereas it did not efficiently target the oligomeric forms exposing multiple single SUMOs (Supplementary Fig. 6c). To test whether a mixture of SUMO1 and SUMO2 moieties was required for targeting, we co-expressed SUMO1-GFP-COMP-HA with FLAG-SUMO2-GFP-COMP-HA and verified that they formed mixed complexes in a FLAG-pulldown experiment (Supplementary Fig. 6b). Also in this setup, no significant targeting by Arkadia was detectable (Supplementary Fig. 6c). While these experiments make the recognition of multi-sumoylated proteins by Arkadia less likely, which is consistent with a preference for substrates marked with SUMO chains, they are insufficient to entirely exclude that other substrates with a different spatial arrangement of multiple mono-SUMOs might be recognized and targeted by Arkadia, similar to what was recently reported for RNF4^31^.

### The Arkadia SOB motif mediates SUMO1 binding

The similarity of the SOB sequence to the SUMO1-binding site in DPP9 (Fig. 2b) prompted us to ask whether this motif contributes to the observed preference of Arkadia towards SUMO1-capped chains. To address this question, we first again employed ITC to compare the affinities of the SUMO-binding domain of Arkadia, with or without mutations in the SOB motif, for linear homogeneous or SUMO1-capped SUMO2 chains (Fig. 2a). Whereas the mutations in the SOB motif (SOB*) (Fig. 2b) had no apparent effect on the binding to SUMO2 chains, they caused a drop in the binding affinity for the SUMO1-capped chains bringing it down to the levels observed for the homogeneous SUMO2 chains (*K*_D_ ~12 µM) (Fig. 2a). These results showed that the SOB motif contributes to specific binding of the SUMO1 moiety in a SUMO1-capped SUMO2 chain. This conclusion was further corroborated by in vivo experiments using yeast cells and GFP test proteins tagged with SUMO chains. While the substrate with a homogeneous 4xSUMO2 chain was targeted even slightly better by Arkadia-SOB* than by its wild-type counterpart (Fig. 2c), the SOB* mutant had lost the preference for the substrate with a SUMO1-capped SUMO chain (Fig. 2d).

### Arkadia SIM1 contributes to SUMO1 binding

**Fig. 3:**
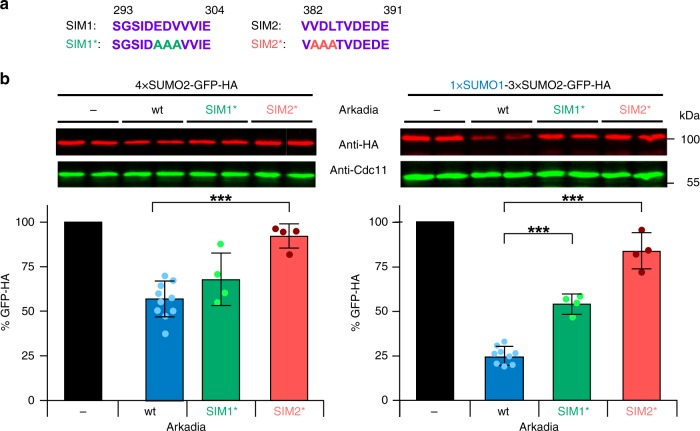
Distinct roles of SIMs in substrate recognition. **a** Depiction of Arkadia SIM sequences and their mutant version (SIM*). The residues that were replaced by Ala residues are highlighted in red or green. **b** Comparison of targeting efficiencies of the same substrates as in Figs. 1 and 2 by wild-type Arkadia and its SIM mutant derivatives. The analysis was carried out as described in Fig. 1c. Bars represent the means. Standard deviations (SD) are indicated by error bars. Quantifications are derived from experiments with 9 independent transformants for wild-type Arkadia (same as in Fig. 1c) and four independent transformants for its SIM* mutant versions. Three asterisks indicate significant differences with *p*-values < 0.001. Statistical significance was determined with unpaired and two-sided Student’s *t*-tests. Source data are provided as a Source Data file

To investigate the roles of the two predicted SIMs in the SUMO-binding domain of Arkadia (Fig. 1a), we generated mutant versions (SIM1* and SIM2*) by exchanging key residues with alanine residues (Fig. 3a). The SIM2* mutation nearly completely blocked activity of Arkadia towards both 4xSUMO2-tagged and SUMO1-capped 3xSUMO2-tagged substrates (Fig. 3b). The SIM1* mutation, by contrast, had no apparent effect on the weak targeting of the 4xSUMO2-tagged substrate, but on the other hand abolished the preference for the substrate tagged with the SUMO1-capped chain in a similar way as the SOB* mutation (Fig. 3b). These data suggest that SIM2 mediates binding of SUMO2 moieties in a chain, whereas SIM1 cooperates with SOB in the recognition of the SUMO1 moiety in a SUMO1-capped SUMO2 chain. The conclusion that two motifs, SOB and SIM1, cooperate in the recognition of a single SUMO1 moiety, whereas SIM2 binds an additional SUMO2 moiety, is consistent with the observation that mutational inactivation of any of the three motifs led to nearly complete loss of targeting of a GFP substrate with a heterodimeric SUMO1–SUMO2 chain (Supplementary Fig. 7).

### Arkadia SOB uses a different binding site in SUMO1 than SIMs

**Fig. 4:**
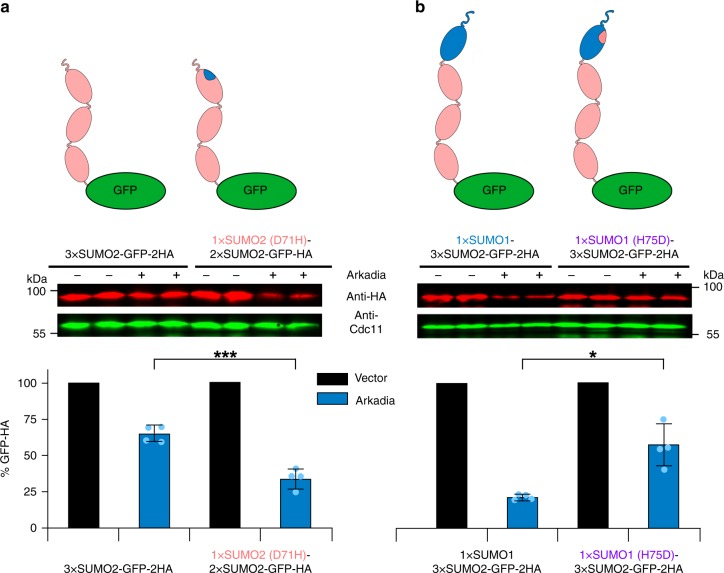
Arkadia SOB binding site in SUMO1 is distinct from that of canonical SIMs. **a** Shown is the analysis of targeting efficiency by wild-type Arkadia upon its co-expression with two otherwise identical 3xSUMO2-GFP substrates differing by the presence of a single amino acid residue exchange (D71H) in the first SUMO2 moiety. **b** Analysis of targeting efficiencies by wild-type Arkadia of otherwise identical SUMO1-3xSUMO2-GFP substrates distinguished by a single amino acid exchange (H75D) in SUMO1. Bars represent the means. Standard deviations (SD) are indicated by error bars. Quantifications are derived from experiments with four independent transformants for each plasmid combination. Asterisks indicate significant differences with *p*-values < 0.02 (one), *p*-values < 0.001 (three). Statistical significance was determined with unpaired and two-sided Student’s *t*-tests. Source data are provided as a Source Data file

Analysis of the binding mode of a SUMO1-specific motif in DPP9, which has considerable similarity to the Arkadia SOB (Fig. 1b), had revealed that it does not bind to the canonical SIM-binding region but instead to a surface of SUMO1 which is far away from the binding site of canonical SIMs on the opposite side of the molecule. This recognition involves a histidine residue at position 75 of SUMO1 (H75), whereas it is blocked by an aspartate residue (D71) at the corresponding position in SUMO2 instead^29^. To investigate whether the Arkadia SOB motif has a similar binding mode as the related DPP9 motif, we converted D71 into histidine (D71H) in the distal SUMO2 of a GFP substrate tagged with a 3xSUMO2 chain, and analyzed the efficiency of its targeting by wild-type Arkadia upon co-expression in yeast cells. The SUMO2-D71H-2xSUMO2-GFP is targeted significantly better by co-expressed Arkadia than 3xSUMO2-GFP, and with a similar efficiency as SUMO1-2xSUMO2-GFP (Fig. 4a and Supplementary Fig. 4). This result indicated that Arkadia SOB utilizes a binding site in SUMO1 similar to the related motif in DPP9. This conclusion was further confirmed by converting H75 in SUMO1 of a SUMO1-capped 3xSUMO chain into aspartate (H75D). In comparison to the original SUMO1-3xSUMO2-GFP substrate, the resulting mutated substrate was targeted relatively poorly by Arkadia, consistent with an important role of the SUMO1 H75 residue for recognition by the Arkadia SOB motif (Fig. 4b). Together, these findings indicated that Arkadia utilizes SIM1 and SOB to recognize different surfaces in the SUMO1 moiety of a SUMO1-capped SUMO2 chain.

### Arkadia-mediated disruption of PML-NBs is SOB-dependent

**Fig. 5:**
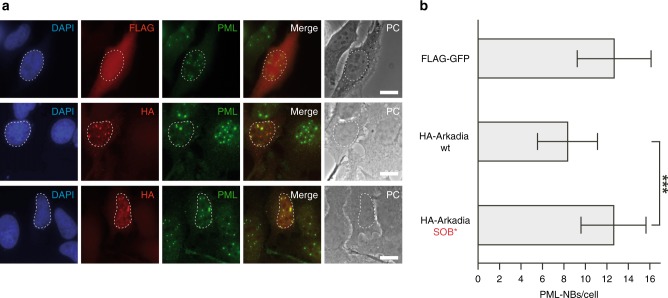
Arkadia-mediated disruption of PML-NBs requires its SOB motif. **a** HeLa cells were transiently transfected with plasmids expressing either FLAG-GFP, wild-type (wt) HA-Arkadia, or its SOB mutant version (SOB*). Cells were fixed, co-stained using mouse anti-FLAG, rabbit anti-PML as well as rat anti-HA antibodies, and analyzed by immunofluorescence microscopy. Nuclei were stained with DAPI. Nuclei of transfected cells positive for HA or FLAG staining are highlighted by dashed lines. Scale bar, 10 µm. **b** Quantitative analysis of the number of PML-NBs in at least 35 cells from each of the samples shown in **a**. ^***^p < 0.001. Bars represent the means. Standard deviations (SD) are indicated by error bars. Statistical significance was determined with unpaired and two-sided Student’s *t*-tests. Source data for **b** are provided as a Source Data file

Another report has indicated that not only RNF4, but also Arkadia contributes to the disruption of PML nuclear bodies (PML-NBs) by targeting sumoylated PML^27^. The PML protein is a known target of SUMO1 modification and its modification with different SUMO paralogs is complex^32–34^. Therefore, we first tested whether transfection of human HeLa cells with a vector overexpressing Arkadia had an effect on PML-NBs. In comparison to control cells transfected with a GFP-expressing vector, cells overexpressing HA-tagged Arkadia displayed a significant reduction in the number of PML-NBs (Fig. 5) similar to what we had previously observed for cells overexpressing RNF4^12,13^. Consistent with a physiological role in the control of PML-NBs, siRNA-mediated Arkadia depletion led to a significant increase in PML-NB number in HeLa and HaCaT cells (Supplementary Fig. 8). Ectopic expression of FLAG-tagged murine Arkadia, which is not or not efficiently targeted by the RNAi designed to specifically target human Arkadia, suppressed the effects of depletion of the endogenous Arkadia (Supplementary Fig. 8). Taken together, our data demonstrate that Arkadia has a role in the regulation of PML-NB abundance. This conclusion is consistent with a report demonstrating that Arkadia mediates SUMO- and ubiquitin-dependent degradation of ectopically expressed GFP-PML protein in HT1080 cells. However, to target GFP-PML in that system, treatment with arsenic trioxide (ATO), which stimulates PML sumoylation, was necessary^27^. Our results, in contrast, show that Arkadia is required to limit the abundance of endogenous PML-NBs also in the absence of ATO, at least in HeLa and HaCaT cells.

Next, we asked whether the SOB motif is required for this function of Arkadia. To address this question, we transfected HeLa cells with the SOB mutant version (SOB*) of Arkadia. In contrast to wild-type Arkadia, which led to a reduction of PML-NBs (see above), Arkadia-SOB* had no effects on the number of PML-NBs (Fig. 5). These data demonstrated that the SOB motif is of critical importance for the role of Arkadia in the control of PML-NB abundance and suggested that targeting of substrates (presumably including PML) marked with SUMO1-capped chains by Arkadia is important for its role in PML-NB regulation.

In order to detect endogenous SUMO1-capped SUMO2-modified proteins and to follow their targeting by Arkadia, we co-expressed 8His-SUMO1 as a translational fusion together with V5-Arkadia, the latter either in its wild-type or RING- or SOB-mutated versions. This approach ensures that 8His-SUMO1 and the different forms of Arkadia are initially produced, after processing by endogenous SUMO proteases, in similar amounts in the stably transfected cells. We then performed Ni pulldowns and analyzed the bound material by anti-SUMO western blotting (Supplementary Fig. 9). While expression of wild-type Arkadia led to a significant reduction of endogenous SUMO1-modified SUMO2-conjugates close to background levels, such conjugates accumulated to much higher levels in cells expressing RING or SOB mutant versions of Arkadia. We conclude that the identified SOB-dependent preference of Arkadia for SUMO1-capped SUMO chains, which was initially identified in our yeast-based experiments using linear SUMO fusions, are valid also for authentic lysine-linked conjugates endogenously produced in human cells.

## Discussion

In this study, we addressed how the second identified human SUMO-targeted ubiquitin ligase Arkadia utilizes its distinct SUMO-binding motifs to select substrates. Using in vitro binding (ITC) and proteolytic targeting assays in a yeast-based in vivo system, and human tissue culture experiments, we characterize Arkadia as a STUbL with a distinct specificity. In contrast to RNF4, which targets substrates with homogeneous SUMO2 chains slightly better than substrates tagged with SUMO1-capped SUMO2 chains, the present work identifies Arkadia as a STUbL that targets substrates with SUMO1-capped SUMO2 chains significantly better than otherwise identical substrates with homogeneous SUMO2 or SUMO1 chains.

The preference for substrates with SUMO1-capped SUMO chains prompted us to search for the relevant binding motif in Arkadia. Strikingly, bioinformatical database searches for motifs related to a SUMO1-specific binding motif in DPP9^29^ yielded Arkadia and Arkadia-like/C18orf25 as the best hits (Fig. 1b). This motif has some similarity to reversed SIMs (type r) such as the one described for RanBP2, which was shown to bind to a SUMO1 surface produced by the beta-strand 2 of SUMO1 and the alpha-helix in a reversed orientation when compared to type b SIMs^35,36^. However, elegant experiments described by Pilla et al. revealed that DPP9 interacts instead with a surface on the opposite side of the SUMO1 molecule, which includes His-75 as a critical residue, whereas an Asp residue in the corresponding position (D71) in SUMO2 inhibits binding^29^. Our in vivo targeting experiments with Arkadia and substrates wherein the respective residues in the distal SUMO moieties of a chain were mutated (SUMO1-H75D and SUMO2-D71H) showed that these changes led to an inversion of targeting efficiency (Fig. 4). These findings demonstrated that the SUMO1-specific sequence in Arkadia uses a surface on SUMO1 distinct from the binding site of canonical SIMs, similar to what had been reported for DPP9. In vitro binding experiments, in addition, had shown for a DPP9-derived peptide, that residue Glu-67 in SUMO1 is critical for binding^29^. In contrast, however, a SUMO1-E67A mutation in a substrate carrying a SUMO1-capped SUMO2 chain had no effect on targeting by Arkadia (Supplementary Fig. 10), indicating that, despite apparent similarities in the binding modes, the related binding motifs of DPP9 and Arkadia bind to SUMO1 slightly differently. To distinguish the identified SUMO1-binding motif in Arkadia from canonical SIMs that bind to a distinct surface of SUMO and to emphasize its paralog-specificity, we refer to it as SOB motif. A nearly identical SOB motif is present in the Arkadia-like protein-1 (ARKL1)/c18orf25 (Fig. 1b), a shorter polypeptide related to the amino-terminal half of Arkadia. The carboxy-terminal half of Arkadia including its RING domain shows similarity to the Arkadia-like protein-2 (ARKL2/RNF165) polypeptide^37^. ARKL2 is encoded in the genome right next to ARKL1 and expressed in the nervous system. ARKL2 has been implicated in the control of neuromuscular connectivity^38^. It is a possibility that ARKL1 and ARKL2 together form a STUbL, which, because of the presence of a SOB, would be expected to exhibit a preference for SUMO1-capped SUMO chains as well.

**Fig. 6:**
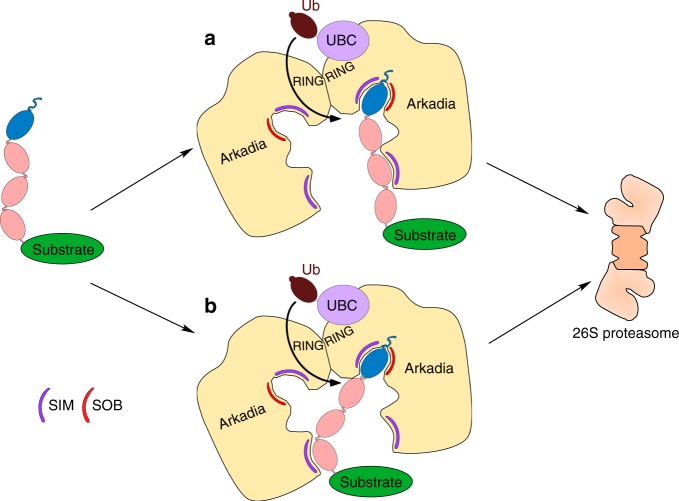
Model of substrate selection by Arkadia. Shown are two modes by which an Arkadia dimer might bind to substrates tagged with SUMO1-capped SUMO2/3 chains and, together with a ubiquitin-conjugating enzyme (UBC), mediate its ubiquitylation and targeting to the proteasome. In **a**, the distal SUMO1 moiety is bound by SIM1 as well as the SOB motif of one of the Arkadia subunits, and a SUMO2/3 moiety in the chain is bound by SIM2 of the same subunit. In **b**, SUMO1 is bound by one Arkadia subunit, whereas a SUMO2/3 moiety in the chain is bound by SIM2 of the other Arkadia subunit. It is also possible that the SIM2 elements of both subunits contribute to binding (not shown). See main text for further details

Arkadia requires two binding motifs, a type r SIM1 and SOB, to recognize a single SUMO1 moiety at the distal end of a SUMO chain (Figs. 2d and  3). The fact that an isolated SUMO-binding domain of Arkadia (residues 285–416) exhibits a preference for binding a dimeric SUMO1–SUMO2 chain (Fig. 2a) suggested that SIM1 and SOB from the same Arkadia polypeptide cooperate in SUMO1 binding (Fig. 6). In addition, a type b SIM (SIM2) is required to recognize at least one additional moiety in a SUMO2 chain. SIM2 was found to be essential for efficient targeting of any of the SUMO-modified substrates tested (Fig. 3 and Supplementary Fig. 7), consistent with the observation that it represents the strongest individual SUMO-binding site of Arkadia^26^. A minimum chain length of three SUMO2s is required for a detectable proteolytic targeting by Arkadia, whereas a dimeric SUMO1–SUMO2 chain is already sufficient to mediate targeting (Supplementary Fig. 4). Targeting improves further with increasing chain length, with trimeric SUMO1-capped chains causing more efficient degradation than dimeric, and tetrameric chains more efficient targeting than trimeric chains (compare Supplementary Fig. 4 and Fig. 1c). Considering the finding that all three identified SUMO-binding motifs are required to recognize one SUMO1 and one SUMO2 moiety (Supplementary Fig. 7), the observed improved recognition of substrates with longer SUMO chains by Arkadia could have multiple reasons. A first explanation is that increasing the distance between the distal SUMO1 cap and an additional SUMO2 moiety in the chain favors simultaneous binding, respectively, of SIM1 together with SOB, and SIM2. A second possibility could be the presence of additional, thus far unrecognized SUMO-binding sites in Arkadia. A third explanation is that binding of substrates carrying SUMO chains involves binding sites in two subunits of an Arkadia dimer. The formation of Arkadia dimers is supported by coprecipitation experiments^27^. An isolated SUMO-binding domain encompassing SIM1, SOB, and SIM2 (residues 285–416) shows similar binding affinity toward dimeric and trimeric SUMO chains in vitro (Fig. 2a), whereas targeting of substrates tagged with trimeric chains by full-length Arkadia in cells is much more efficient than that of substrates tagged with dimeric chains. This observation argues against the first explanation, at least for a single SUMO-binding domain. The complete disruption of targeting caused by mutation of SIM2 argues against the existence of additional relevant binding sites that might cooperate with SIM1 and SOB in recognizing SUMO chains, although this result does not completely rule out the possibility of additional weak SUMO-binding sites in Arkadia. To explain the data, we therefore favor the third explanation according to which SIM1 and SOB together recognize the SUMO1 cap, and the two SIM2 motifs of an Arkadia dimer provide additional binding to SUMO2 moieties in a chain. The observation that tetrameric chains cause a significantly more efficient substrate targeting than trimeric ones could then again be explained by a more favorable spatial arrangement of SUMO moieties that facilitates spanning of a chain from the SUMO-binding domain of one Arkadia subunit to the second one in the other subunit (Fig. 6). These considerations are consistent with interpretations of data obtained from comparable experiments performed with RNF4, which displays a similar chain length dependence as Arkadia, albeit without a preference for SUMO1-capped chains^13–15^.

Arkadia’s preference for substrates tagged with SUMO1-capped chains raises the question when and how this type of SUMO modification is used in cells. The current understanding of the role of mixed SUMO chains is still very limited. Systematic analyses of SUMO proteomes have identified SUMO1–SUMO2/3 linkages in cell extracts^18^. These studies, however, do not provide information regarding specific substrates carrying mixed SUMO chains and the exact topology of so-modified proteins. It is also presently unknown how the relative amounts of homogeneous SUMO2/3 chains versus SUMO1-capped chains on specific substrates, or in general, change in response to physiological cues. It is known that the formation of SUMO2/3 conjugates is strongly stimulated by various stress conditions (see “Introduction”). Similar to the close ubiquitin relative Rub1/Nedd8, which was observed to act as a terminator of ubiquitin chains^39^, SUMO1 appears to act as a terminator of SUMO2/3 chains because of its low propensity to serve as an acceptor for the attachment of additional SUMO moieties^17,18^. The availability and frequency of conjugation of the different paralogs, SUMO1 versus SUMO2/3, is therefore probably a critical determinant of the SUMO chain length in cells. This notion is supported by in vitro experiments showing that the length of polymeric SUMO chains could be influenced by changing the relative amounts of SUMO paralogs^18^. Other parameters that will contribute to the control of SUMO conjugates are deconjugating enzymes with distinct paralog specificities as well as SUMO-binding proteins including STUbLs^7,40–42^. In cells, the conjugation rates for SUMO2/3 are different in unstressed versus stressed cells, as well as during the period in which a stress response is declining again, and also during the cell division cycle^12,19,43,44^. As a consequence, not only fewer SUMO2/3 conjugates, but also ones with shorter chains should be relatively more frequent in nonstressed cells or in cells recovering from a stress condition. Multiple reasons for a requirement of the preference for SUMO1-capped chains observed for Arkadia can be envisioned. Arkadia may have a role in the degradation of substrates with very short SUMO chains because SUMO1-capped chains as short as dimers are sufficient for targeting by Arkadia, but not for recognition by RNF4. This function may be particularly important when SUMO2 conjugation is low or downregulated. Another possibility is that SUMO1-capped SUMO2 chains not only serve as a degradation signal but also guide so modified conjugates to specific sites in the cell, where they are eventually downregulated again by Arkadia. Physiological evidence for SOB-dependent targeting by Arkadia stems from our analysis of PML nuclear bodies (NBs), the numbers of which are influenced both by endogenous levels of Arkadia as well as by ectopic overexpression (Fig. 5 and Supplementary Fig. 8)^27^. Strikingly, however, the latter is abrogated by mutational inactivation of the SOB motif suggesting that targeting of substrates carrying SUMO1-capped SUMO chains are the relevant targets (Fig. 5). A likely substrate is the PML protein, which is modified both with SUMO1 and SUMO2, and forms the scaffold of PML-NBs^32–34,41^. Additional experiments with human cells co-expressing Arkadia and 8His-SUMO1 showed that a significant amount of proteins with authentic apparently lysine-linked hybrid SUMO1–SUMO2 modification are present in these cells, and that these proteins are downregulated by Arkadia in a SOB-dependent manner (Supplementary Fig. 9).

For Arkadia it has been shown that the SUMO-binding domain, including SIM1 and SOB, contributes to the localization of Arkadia to Polycomb bodies in the nucleus and to the role of Arkadia in the regulation of transcription^37^. Arkadia was also shown to regulate the release of Xeroderma pigmentosum C (XPC) from UV-damaged DNA in a SUMO-dependent manner after initiation of nucleotide excision repair^28,45^. Interestingly, Arkadia cooperates with the Ubc13-Mms2 ubiquitin-conjugating enzyme to mediate UV-induced modification of XPC with K63-linked ubiquitin chains, which do not promote degradation by the proteasome. It will be interesting to investigate whether an interaction of Arkadia with SUMO1-capped chains is involved in these processes, or whether SOB-dependent targeting of substrates generally occurs via other ubiquitin-conjugating enzymes such as UbcH5 (known to cooperate with Arkadia^27,28^), leading to different ubiquitin chain types (such as K48-linked ones) that typically promote degradation by the proteasome. Yet another possible scenario was recently described for the transcription factor Nrf2. K48-linked ubiquitylation of Nrf2 by Arkadia appears to result in its stabilization rather than its proteasomal degradation^46^. Whether the SOB-dependent mode of recognition of composite SUMO1–SUMO2 modifications determines such different downstream fates of substrates, remains to be explored. While our experiments with monomeric and oligomeric substrates suggested that Arkadia preferentially binds SUMO polymers, it cannot be excluded that it also targets multi-mono-sumoylated proteins with distinct topologies or mono-sumoylated substrates with additional binding sites for Arkadia. In any case, the recognition of a composite signal made up by two SUMO paralogs (SUMO1 and SUMO2/3) represents another example of diversification of the code produced by ubiquitin family (UbF) modifiers. An earlier example of decoding a composite signal of UbF modifiers was provided by the RAP80 protein, which simultaneously binds ubiquitin and SUMO moieties via UIM and SIM sequences^47,48^.

## Methods

### Analysis of Arkadia-mediated targeting in *S. cerevisiae*

GFP reporter proteins with linear N-terminal SUMO chains were co-expressed in *S. cerevisiae* from centromeric plasmids with plasmids expressing Arkadia. Yeast strains used were JD47–13C (wild-type), its *pdr5∆* derivative YGA34, or its *slx5∆ uls1∆* derivative YKU121^5^. The constructs used in this study are listed in Supplementary Table 1 and were generated employing PCR reactions and the oligonucleotides listed in Supplementary Table 2. All constructs were verified by sequencing. Mutations were introduced into Arkadia- or SUMO-encoding DNA sequences by overlap extension PCR. For quantitative Western blot analyses, cells expressing the GFP-HA tagged SUMO1/2 substrates were transformed with plasmids expressing Arkadia or with an empty vector. Cells were grown to log phase (OD_600_ of 0.6–0.8) in the presence of 100 µM copper sulfate. 3.5 OD_600_ units (corresponding to 3.5 mL of a culture with OD at 600 nm of 1) were harvested and boiled protein extracts were made using 175 µL of loading buffer (0.0625 M Tris/HCl pH 6.8, 2% SDS, 10% glycerol, 0.002% bromophenol blue, and 100 mM DTT). Proteins were separated by 10% SDS-polyacrylamide gel electrophoresis and transferred to nitrocellulose membranes by semidry blotting. The SUMO-GFP-HA substrates and an internal loading control (Cdc11) were detected, respectively, with mouse mono-clonal anti-HA (16B12; Covance MMS-101R, diluted 1/1000) and rabbit poly-clonal anti-Cdc11 (Santa Cruz Biotechnology SC-7170, diluted 1/5000) primary and fluorophore-coupled secondary anti-mouse 680 (Rockland, 610-144-002) and anti-rabbit 800 (Rockland, 611-145-002-0.5) antibodies (both diluted 1/5000) and the Odyssey Infrared Imager (LI-COR Biosciences). Uncropped scans are available for all the blots in the Source Data file.

### Pulse chase analysis

To monitor protein turnover, cells from cultures harboring 40 OD_600_ units were harvested in two tubes by centrifugation and each pellet was resuspended in 25 mL of 2× minimal medium lacking methionine and containing 100 μM CuSO_4_ for induction of expression. After addition of 10 μL of S^35^ labeled methionine (100 µCi, Hartmann Analytic: 179352), cells were incubated for a pulse period of 5 min at 30 °C. The cells were then collected by centrifugation and resuspended in 25 mL of 2× minimal medium containing 100 μM CuSO_4_ and 10 mM cold methionine. One sample was treated with 20 μM MG132. Cells were harvested either immediately or after chase periods of 7.5, 15, or 30 min. Protein extracts were generated using glass beads in lysis buffer (50 mM HEPES pH 7.5, 5 mM EDTA pH 8.0, 150 mM NaCl, and 1% Triton X-100). The SUMO-GFP-HA substrates were pulled down using anti HA agarose beads (Roche, 11815016001), eluted with 1× loading buffer and run on a 10% SDS-polyacrylamide gel, transferred onto a nitrocellulose membrane and analyzed using a Molecular Dynamics Storm 860 Phosphorimager.

### Immunoprecipitation and detection of ubiquitylation

Precipitation of FLAG-tagged SUMO-GFP substrates and detection of their ubiquitylation was carried out as follows: Cells from cultures harboring 15 OD_600_ units were collected by centrifugation and frozen in liquid nitrogen. The pellets were then suspended in 1.85 N NaOH, 7.4% β-mercaptoethanol and incubated on ice for 10 min. The samples were centrifuged for 10 min at 16,000 × *g* at 4 °C. The supernatant was discarded, the pellet washed with acetone and resuspended in 500 μL TSG buffer (0.5 M Tris, 6.5% SDS, 12% glycerol, 100 mM DTT). 390 μl of an extract were diluted with 13 mL RIPA buffer (50 mM Tris/HCl pH 7.4, 150 mM NaCl, 0.05% Triton X100, 50 mM NEM, proteinase inhibitors (Roche)). After addition of 50 μL anti-FLAG resin (Sigma, F2426), binding was allowed to occur overnight at 4 °C. The beads were then washed thrice with 1 mL RIPA buffer, resuspended in 200 μL of LLB (0.0625 M Tris/HCl pH 6.8, 2% SDS, 10% glycerol, 0.002% bromophenol blue) and incubated for 15 min at 37 °C. The samples were then passed through a 0.45 μm filter. 100 mM DTT was added to the filtrate followed by boiling for 5 min. The samples were then loaded on 10% SDS-polyacrylamide gels and analyzed by anti-ubiquitin Western blotting with P4D1 mono-clonal anti-ubiquitin antibody (diluted 1/2500; Santa Cruz Biotechnology, SC-8017).

### Fluorescence microscopy

Analysis of GFP fusion proteins was performed using a Zeiss Axioplan microscope. DNA was stained with 0.5 µg per mL 4′,6-diamidino-2-phenylindole (DAPI; PanReac AppliChem, A1001) after treating cells with 10% ethanol for 10 min.

### Constructs for in vitro binding experiments

For expression in *E. coli*, constructs encoding linear poly-SUMO chains were generated as follows: After the combined PCR/ligation reaction, the 2xSUMO2-ΔN11 or 3xSUMO1-ΔN15 DNA polymers were separated from monoSUMO-encoding fragments by agarose gel electrophoresis, and individually extracted using NucleoSpin columns (Macherey-Nagel, 740609.250). These were then subjected to a second PCR reaction with primers to create BamHI and XhoI restriction sites, a C-terminal GG motif and a stop codon. The resulting fragments coding for di- and tri-SUMO2-ΔN11 chains were amplified and cloned into pGEX-4T2. Mixed SUMO chains were made of the previous derived constructs. MonoSUMO1ΔN15 was amplified by PCR (BamHI/BglII) and cloned into dephosphorylated pGEX-4T2-1xSUMO2 or pGEX-4T2-1xSUMO2 by restriction digest using BamHI. Orientation was checked via sequencing. Expression from these plasmids resulted in GST-tagged artificial SUMO2-ΔN11 or SUMO1-ΔN15 monomers or linear-linked chains with up to three monomers. Each monomer in these chains is separated from the other by Arg-Ser replacing the natural Gly–Gly motif. A clone coding for murine Arkadia was kindly provided by Saitoh^45^. The SIM domain coding for amino acids 285–416 was amplified by PCR and subcloned into pGEX-TEV/N (a modified pGEX-4T2 vector carrying a TEV protease cleavage site after the thrombin cleavage site).

### Protein purification and isothermal titration calorimetry

The linear polySUMO1 and poly-SUMO2 proteins as well as the Arkadia construct were purified as GST-fusion proteins from *E. coli* Rosetta™ 2 (DE3) pLysS (Novagen) cells cultivated in LB-medium with ampicillin and chloramphenicol. Expression of the recombinant proteins was induced by addition of 0.1 mM IPTG at 20 °C. After harvesting by centrifugation, cells were lysed by sonication in GST-buffer I (50 mM Tris/HCl, pH 7.5, 150 mM NaCl, 2 mM DTT) with 0.1% Triton-X-100 and in the presence of protease inhibitors. After clarification of the lysate by centrifugation for 1 h at 50.000 × *g,* all GST-fusion proteins were purified from bacterial extracts by incubation of the supernatant with glutathione-agarose beads (ProtinoGST 4B, Macherey-Nagel), followed by extensive washing with GST-buffer I with 5% glycerol (and 0.1% Triton X-100 in the case of tetraSUMOs, and Arkadia SIM mutants) and GST-buffer I with 300 mM NaCl. Furthermore, the glutathione-agarose beads with the attached proteins were incubated for 30 min with a buffer to deplete bacterial chaperones (50 mM Tris/HCl pH 7.5, 150 mM NaCl, 60 mM KCl, 10 mM MgCl_2_, 2 mM ATP, and 2 mM DTT). Afterwards the fusion proteins were cleaved in GST-buffer I by incubation for 1 h with thrombin/TEV at RT or overnight at 4 °C. The supernatant was applied to Q-Sepharose FF (GE Healthcare) and eluted with a gradient from 50 to 1000 mM NaCl over seven column volumes. The fractions with the target proteins were analyzed by SDS-PAGE and further purified by gel filtration on Superdex 75 (GE Healthcare) in ITC buffer (50 mM Tris/HCl, pH 7.5, 150 mM NaCl, 2 mM DTT, 5% glycerol). Protein concentrations were determined by measuring absorption at 280 nm. Binding of SUMOs to Arkadia was investigated by isothermal titration calorimetry using an iTC200 (GE Healthcare). All experiments were performed in ITC buffer at 30 °C unless otherwise stated. The proteins were injected from a syringe in 20–30 steps up to a twofold to threefold molar excess. Concentrations were chosen so that the binding partners in the cell were at least fivefold higher than the estimated dissociation constant, if possible. The ligands in the syringe were again at least tenfold more concentrated. Titration curves were fitted to the data using ORIGIN (supplied by the manufacturer) yielding the stoichiometry N, the binary equilibrium constant *K*_A_ ( = *K*_D_^−1^) and the enthalpy of binding Δ*H*. The entropy of binding Δ*S* was calculated from the relationship Δ*G* = −*RT*•ln*K*_A_ and the Gibbs–Helmholtz equation. The values were averaged from three to four titrations.

### Immunohistochemistry

HaCaT and HeLa B cells (HeLa B cells: European Collection of Authenticated Cell Cultures (ECACC) Catalog No.: 85060701, HaCaT, Flp-InTM T-RExTM 293 cell line (Thermo Fisher Scientific Cat. R789007) were grown on coverslips and fixed in PBS with 3% para-formaldehyde. After permeabilization in PBS with 0.2% saponin (Sigma–Aldrich), cells were blocked in PBS, 3% BSA and 0.2% saponin. Primary and secondary antibodies (Invitrogen) were applied in blocking buffer. Coverslips were embedded in ProLong^®^ Gold Antifade (Invitrogen) and examined using a Zeiss Axioplan2 fluorescence microscope. Proteins and epitope tags were detected with rabbit anti-PML (A167 and A168 diluted 1/100; Bethyl), rat anti-HA (3F10, diluted 1:250; Roche 11867423001) and mouse anti-FLAG (M2, diluted 1/500; Sigma–Aldrich, F3165) primary antibodies. The secondary antibodies were labeled with Alexa Fluor^®^ 546 or Alexa Fluor^®^ 647 (Invitrogen A-11030 and A32733) and diluted 1/1000.

### Analysis of SUMO1–SUMO2 hybrid conjugates in human cells

8His-SUMO1-V5-Arkadia (either wild-type, or mutated in the RING or SOB motifs) were cloned by inserting an 8His-SUMO1-V5 sequence and the different Arkadia ORF versions into a modified pcDNA5/FRT/TO plasmid (Invitrogen)^49^. The resulting plasmids were integrated into Flp-In™ T-REx™ 293 cell line (Thermo Fisher Scientific) according to the manufacturer’s recommendations to simultaneously express 8His-tagged SUMO1 and V5-tagged Arkadia variants. 1.5 million cells either expressing 8His-SUMO1 alone or as a translational fusion along with V5-Arkadia wild-type, RING* and SOB* mutant versions were grown in 10 cm^2^ petri dishes for 14 h. Expression of tagged proteins was induced by addition of 1 µg per mL doxycycline for 29 h. After removing the media, the cells were scraped off the plates in 250 µL 1.85 N NaOH, 7.4% β-mercaptoethanol. The samples were sonicated, incubated on ice for 10 min, before 350 µL 55% TCA was added and incubation on ice continued for 15 min. After centrifugation, the pellets were washed once with 1 ml of 100% acetone. The pellets were dissolved in 200 µL TSG buffer (0.5 M Tris, 6.5% SDS, and 12% glycerol). The samples were vortexed briefly and incubated at 65 °C for 20 min. 180 µL of the protein extracts were bound to prewashed 100 µL of Ni-NTA beads. The volume was adjusted to 1.25 mL using binding buffer (8 M urea, 100 mM Tris pH 8). Binding was performed at room temperature for 2 h. The beads were then washed thrice with binding buffer, resuspended in 100 µL DTT containing 1xLDS buffer (Invitrogen, NP0007) and boiled for 5 min at 95 °C. Samples were then loaded onto gradient gels (Invitrogen, NP0322) and analyzed by western blotting using anti-SUMO1 (diluted 1/100, derived from hybridoma cell line clone 71-2-1), anti-SUMO2 (diluted 1/100, derived from hybridoma cell line 8A2) (both cell lines were kind gifts from Dr. Frauke Melchior), mouse anti-V5 (R960-25, diluted 1/2500; Invitrogen, P/N 46-1157) primary and anti-mouse (Goat anti-mouse IgG secondary antibody, diluted 1/5000; Pierce, 62-6520) or anti-rabbit (Goat anti-Rabbit IgG secondary antibody, diluted 1/5000; Pierce, 65–6120) secondary antibodies

### Statistical analyses

The bar graphs show averages of several independent experiments (see legends for details) and the standard deviation of the means. Statistical significance was determined with unpaired and two-sided Student’s *t*-tests.

### Reporting summary

Further information on research design is available in the Nature Research Reporting Summary linked to this article.

## Supplementary information

Supplementary information

Reporting summary

Source Data

## Data Availability

The source data for blots and bar graphs underlying Figs. 1c, d, 2a, c, d, 3b, 4, 5b, and Supplementary Figs 2, 4, 5, 6, 7, 8c, d, 9 and 10 are provided as a Source Data file. A reporting summary for this article is available as a Supplementary Information file. All other data are available from the corresponding authors on reasonable request.
