# Supplementary material for: Arkadia/RNF111 is a SUMO-targeted ubiquitin ligase with preference for substrates marked with SUMO1-capped SUMO2/3 chain
Source: Nat Commun. 2019 Aug 15;10:3678. doi: 10.1038/s41467-019-11549-3 (PMC6695498; doi:10.1038/s41467-019-11549-3)
Supplement: Supplementary file 1 — Supplementary information [file 41467_2019_11549_MOESM1_ESM.pdf]

SUPPLEMENTARY INFORMATION

Arkadia/RNF111 is a SUMO-targeted ubiquitin ligase with preference for substrates marked with SUMO1-capped SUMO2/3 chain

Sriramachandran et al.

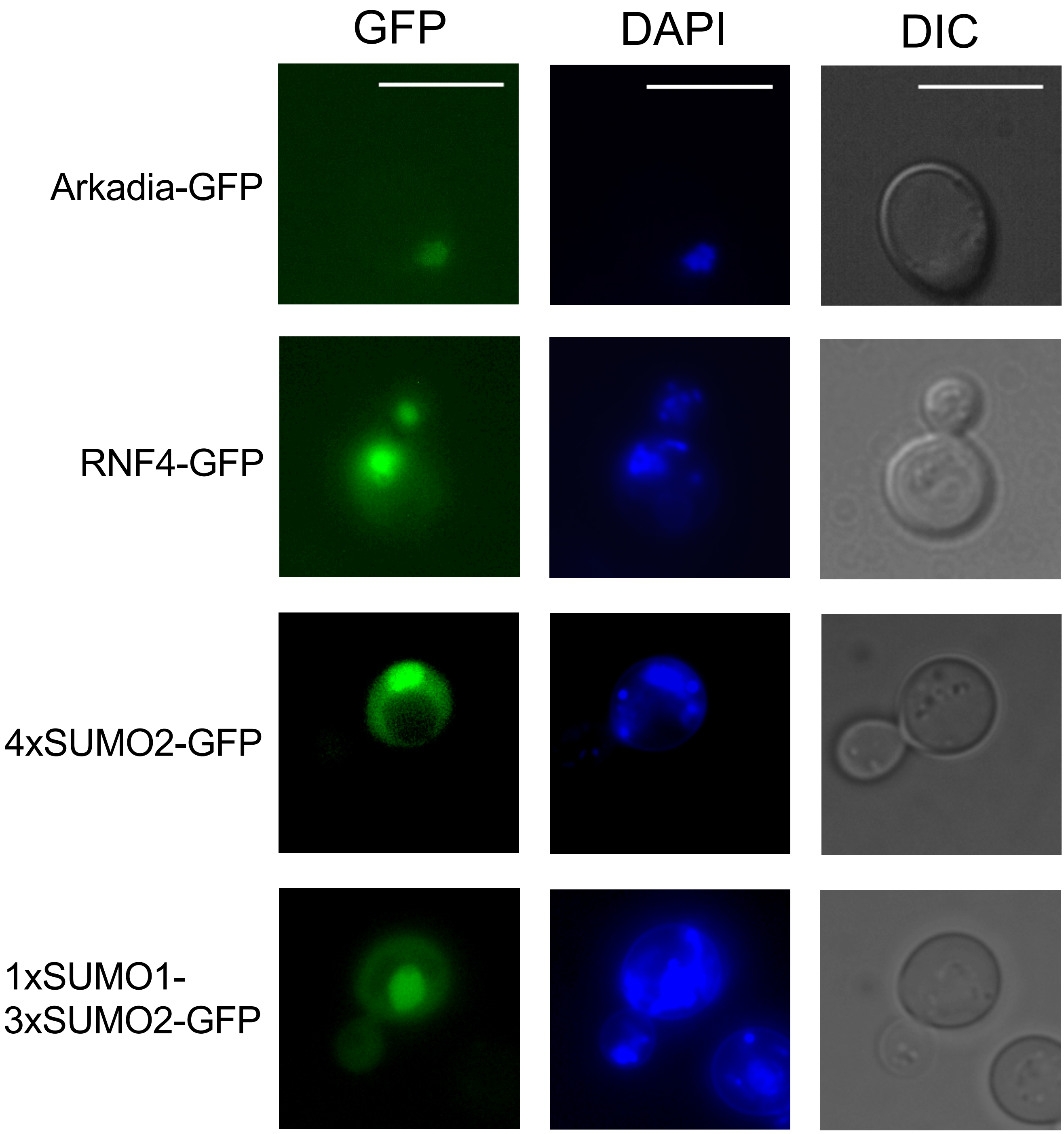

Supplementary Fig. 1

Localization of STUbLs and model substrates in yeast. *S. cerevisiae cells* were transformed with plasmids expressing the indicated GFP fusion proteins and analyzed by fluorescence and differential interference contrast (DIC) microscopy after staining with 4′,6-diamidino-2-phenylindole (DAPI). Because of relatively low abundance, Arkadia-GFP was expressed in *uba1-ts26* cells, which are deficient in ubiquitin-dependent proteolysis due to a defect in ubiquitin-activating enzyme. The other constructs were expressed in wild-type cells. Scale bar, 10 μm.

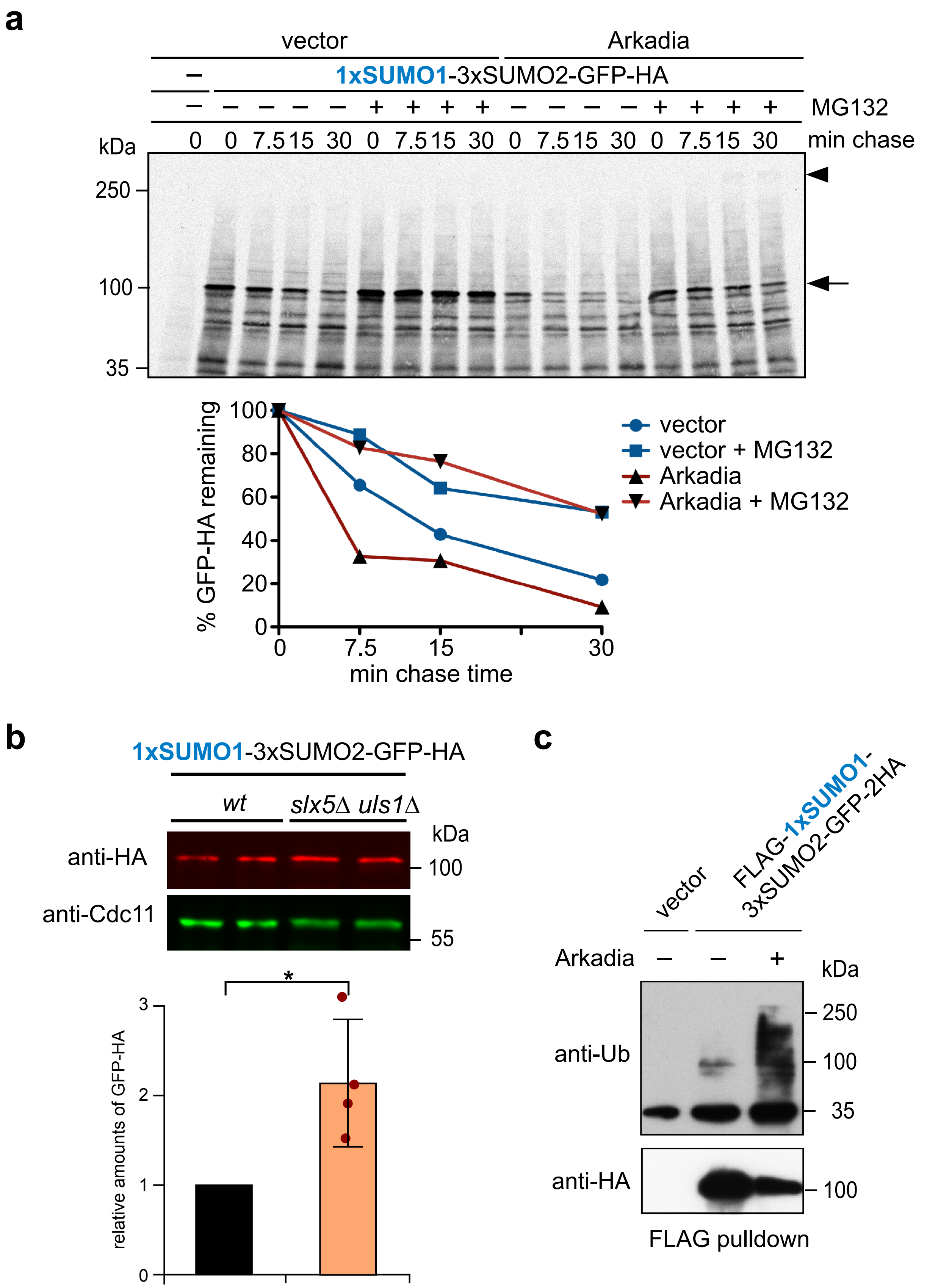

Supplementary figure 2. Arkadia promotes ubiquitylation and degradation of substrates with SUMO1-capped SUMO2 chains by the proteasome. (Legend on next page).

Supplementary Fig. 2

Arkadia promotes ubiquitylation and degradation of substrates with SUMO1-capped SUMO2 chains by the proteasome. a Pulse chase analysis of cells co-expressing the indicated GFP substrate fused to a linear SUMO1- capped 3xSUMO2 chain either with an empty vector or with Arkadia. Two exponential cultures were split. One half was used to perform the pulse chase experiment in the absence, and the other half in the presence of 20 µM MG132. An arrow indicates the position of the unmodified GFP substrate. An arrowhead points to an accumulation of high-molecular weight, likely ubiquitylated forms of the substrate detectable in higher amounts upon co-expression of Arkadia and after MG132 treatment. Below the autoradiography, the results from a phosphor-Imager quantification of the signals are plotted. **b** Analysis of levels of SUMO1-3xSUMO2-GFP substrate either in wild-type yeast cells or its *slx5*Δ *uls1*Δ mutant derivative lacking the two known yeast ULS/STUbL enzymes by quantitative Western blotting was carried out as described in the legend to Figure 1C. Bars represent the means of data obtained with independent transformants (n=4). Standard deviations (SD) are indicated by error bars. Asterisks indicate significant differences with p-values < 0.1. Statistical significance was determined with unpaired and two-sided Student's t-tests. **c** Arkadia promotes the ubiquitylation of a GFP-substrate carrying a SUMO1-capped 3xSUMO2 chain. An N-terminally FLAG-tagged variant of the substrate was co-expressed either with an empty vector or with Arkadia. Extracts were prepared under denaturing conditions followed by a pulldown using an anti-FLAG antibody resin. Bound material was analysed by anti-HA Western blotting to detect the amount of recovered GFP-HA substrate, and by anti-ubiquitin blotting to detect ubiquitylated forms of the substrate. Note the much higher level of ubiquitylated forms of the substrate upon co-expression of Arkadia despite a lower steady state level of the substrate due to Arkadia-mediated degradation. Source data for **a**, **b**, and **c** are provided as a Source Data file.

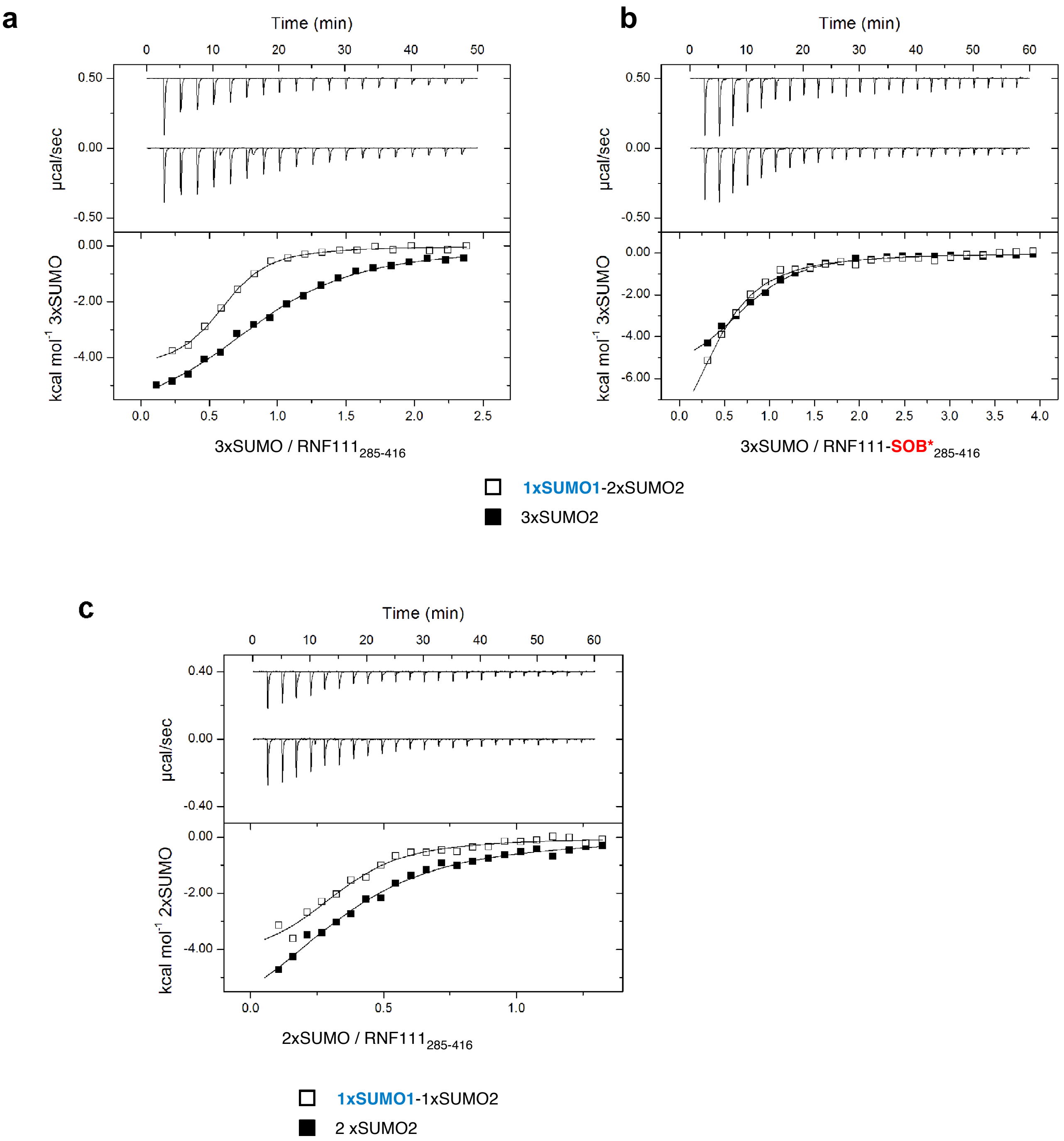

Supplementary Fig. 3

Affinity of the Arkadia SUMO-binding domain to SUMO chains measured by isothermal titration calorimetry. **a** Calorimetric titration of the Arkadia SUMO-binding domain with trimeric linear SUMO chains. Shown are the profiles of typical titrations of the indicated trimeric SUMO chains into a solution of $\text{Arkadia}_{285\text{-}416}$ (upper panel, top to bottom) and the integrated data, normalized to the concentration of the SUMOs (lower panel). **b** Same as in **a**, but with the SOB* mutant version (see Figure 2a) of the Arkadia SUMO-binding domain. **c** Same as in **a** but with dimeric SUMO chains.

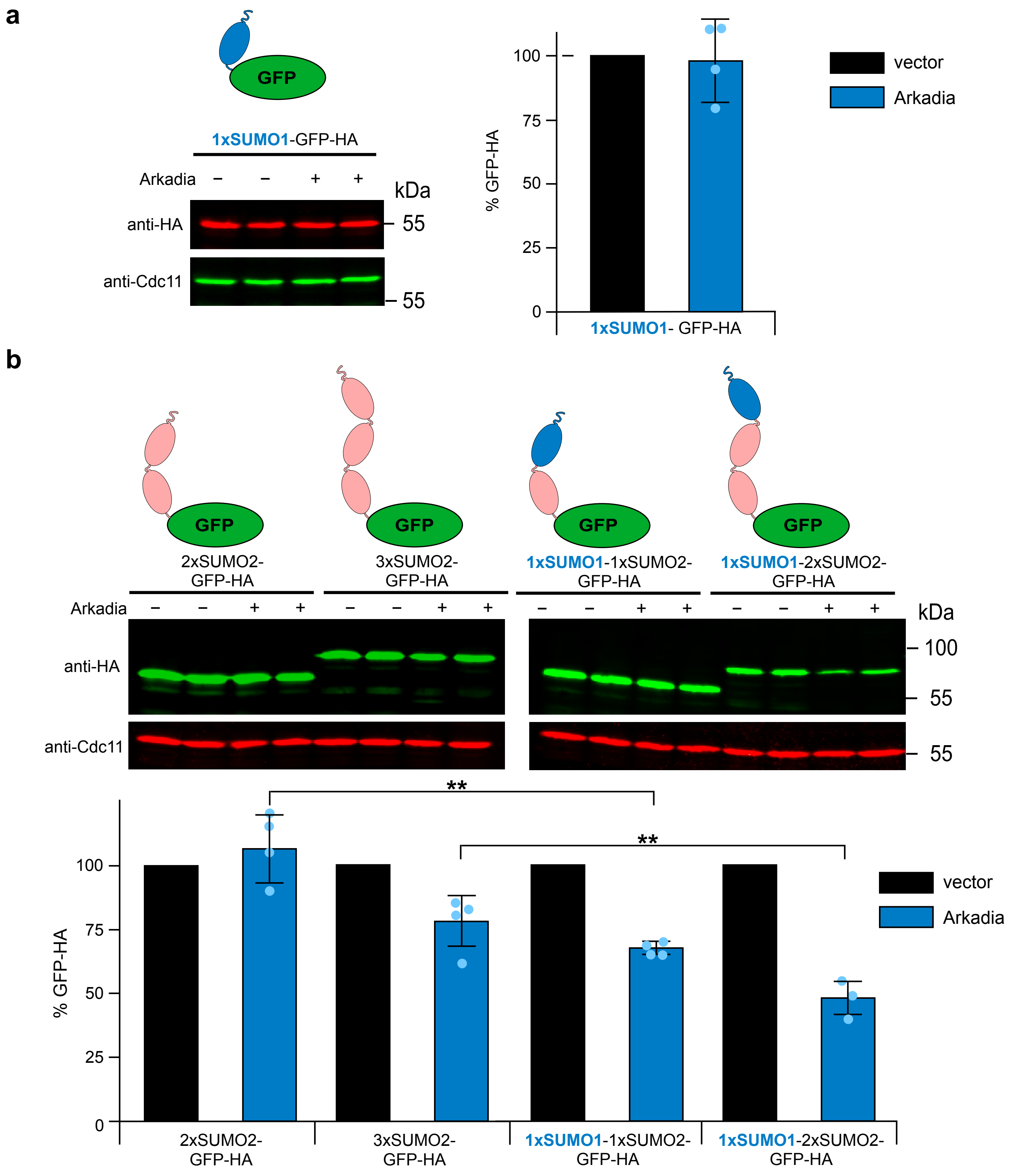

Supplementary Fig 4

SUMO chain type and length preference of Arkadia. (legend on next page)

Supplementary Fig. 4

SUMO chain type and length preference of Arkadia. a Quantitative Western blot analysis showing that Arkadia does not target a SUMO1-GFP test protein upon co-expression in yeast cells. **b** Quantitative Western blot analyses addressing the chain length preference of Arkadia with substrates carrying either homogeneous SUMO2 chains (left panel) or SUMO1-capped SUMO2 chains (right panel). Arkadia does not target a 2xSUMO2-GFP test protein upon expression in yeast cells, and only weakly targets a 3xSUMO2-GFP variant of the substrate (left panel). In contrast, significant targeting of SUMO1-SUMO2-GFP is already detectable, and targeting efficiency increases with chain length. Western blot analysis for **a** and **b** was carried out as described in the legend to Figure 1c. Bars represent the means of data obtained with independent transformants (n=4). Standard deviations (SD) are indicated by error bars. Two asterisks indicate significant differences with p-values < 0.01. Statistical significance was determined with unpaired and two-sided Student's t-tests. Source data for **a** and **b** are provided as a Source Data file.

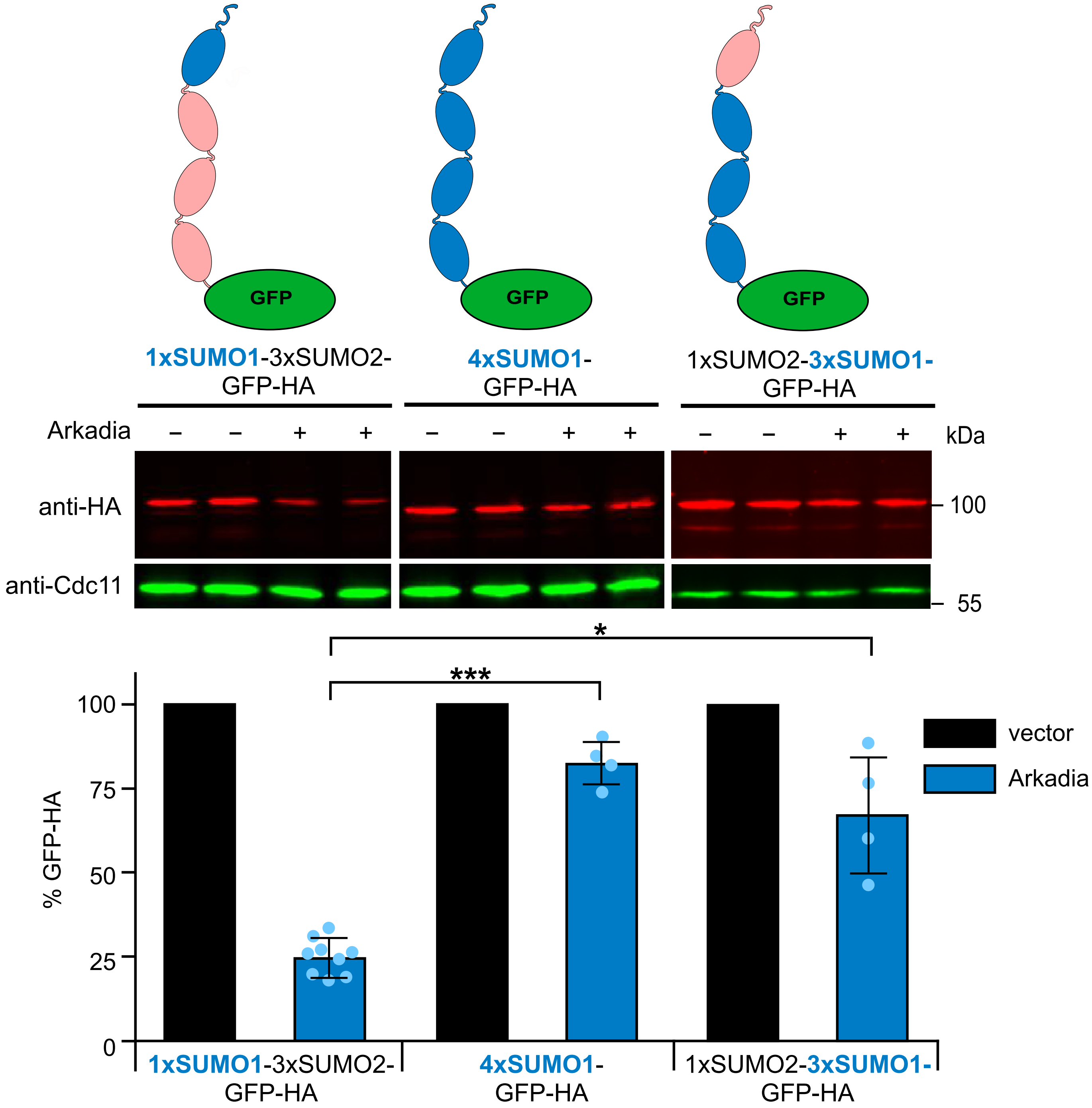

Supplementary Fig. 5 (legend on next page)

Supplementary Fig. 5

Arkadia has a preference for SUMO1-capped SUMO2 chains. Shown is a comparative analysis of targeting efficiency of GFP-HA substrates carrying distinct linear tetra-SUMO chains upon co-expression of Arkadia in *S. cerevisiae*. Levels of each indicated data point for the distinct 4xSUMO-HA substrates were determined with independent transformants by quantitative anti-HA Western blotting as described in the legend to Figure 1c. Data for 1xSUMO1-3xSUMO2-GFP-HA are the same as the ones shown in Figure 1c (n=9). For 4xSUMO1-GFP-HA and 1xSUMO2-3xSUMO1-GFP-HA, n=4. Bars represent the means of data obtained with independent transformants. Standard deviations (SD) are indicated by error bars. An asterisk indicates significant differences with p-values < 0.02, three asterisks: p < 0.001. Statistical significance was determined with unpaired and two-sided Student's t-tests. Source data are provided as a Source Data file.

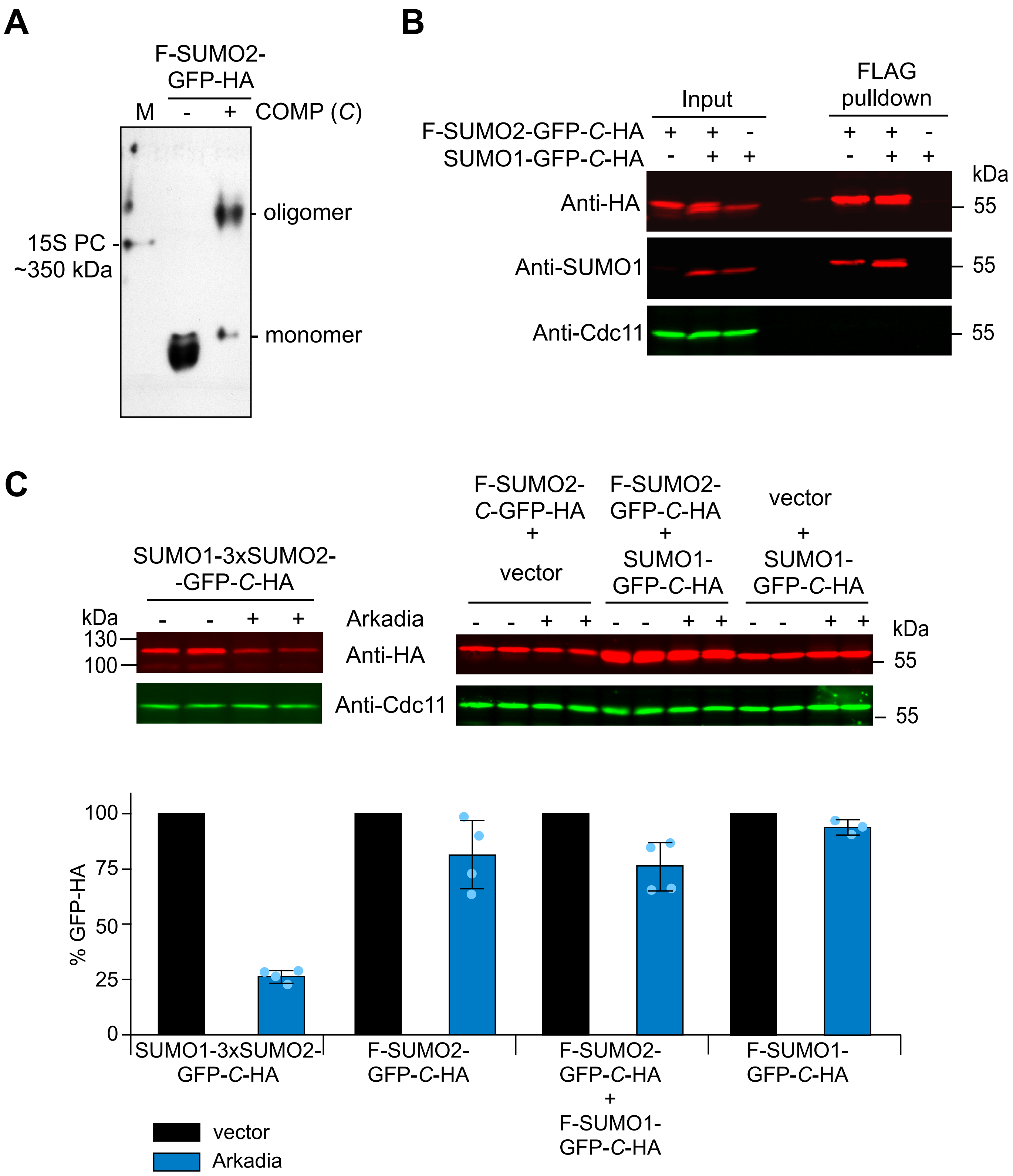

Supplementary figure 6. Arkadia does not efficiently target oligomeric GFP-HA substrates presenting multiple mono-SUMO1 or mono-SUMO2 fusions or combinations of the two (Legend on next page).

Supplementary Fig. 6

Arkadia does not efficiently target oligomeric GFP-HA substrates presenting multiple mono-SUMO1 or mono-SUMO2 fusions or combinations of the two. **a** SUMO2 forms an oligomer when fused to a COMP domain. Shown is a native gel analysis of extracts from yeast cells expressing FLAG- SUMO2- GFP-HA either with (+) or without (-) a pentamerization domain of mouse cartilage oligomeric matrix protein COMP (*C*) inserted between GFP and HA. Positions of the monomeric and oligomeric forms are indicated. The 15S proteasome precursor complex with a mass of ~350 KDa is run on the same gel for size comparison [1]. **b** Anti- FLAG pulldown experiment with yeast extracts showing that F-SUMO2-GFP-*C*-HA co-precipitates and thus forms hetero-oligomeric complexes with SUMO1-GFP-*C*-HA. Cells were grown to log phase and protein extracts and FLAG immunoprecipitations were performed under native conditions. Input and eluate samples were analyzed by anti-HA and anti-Cdc11 western blotting. Endogenous Cdc11 protein levels were detected as a loading control. **c** Cells expressing either SUMO1-3xSUMO2-GFP-*C*-HA (left panel), FLAG- SUMO2-GFP-*C*-HA or SUMO1-GFP-*C*-HA, or the latter two together (right panel), were transformed either with an empty vector or a plasmid expressing Arkadia. Cells were grown to log phase and protein crude extracts were prepared and analysed by quantitative western blotting. Bars represent the means of data obtained with independent transformants (n=3 or 4). Standard deviations (SD) are indicated by error bars. Source data for **a**, **b**, and **c** are provided as a Source Data file.

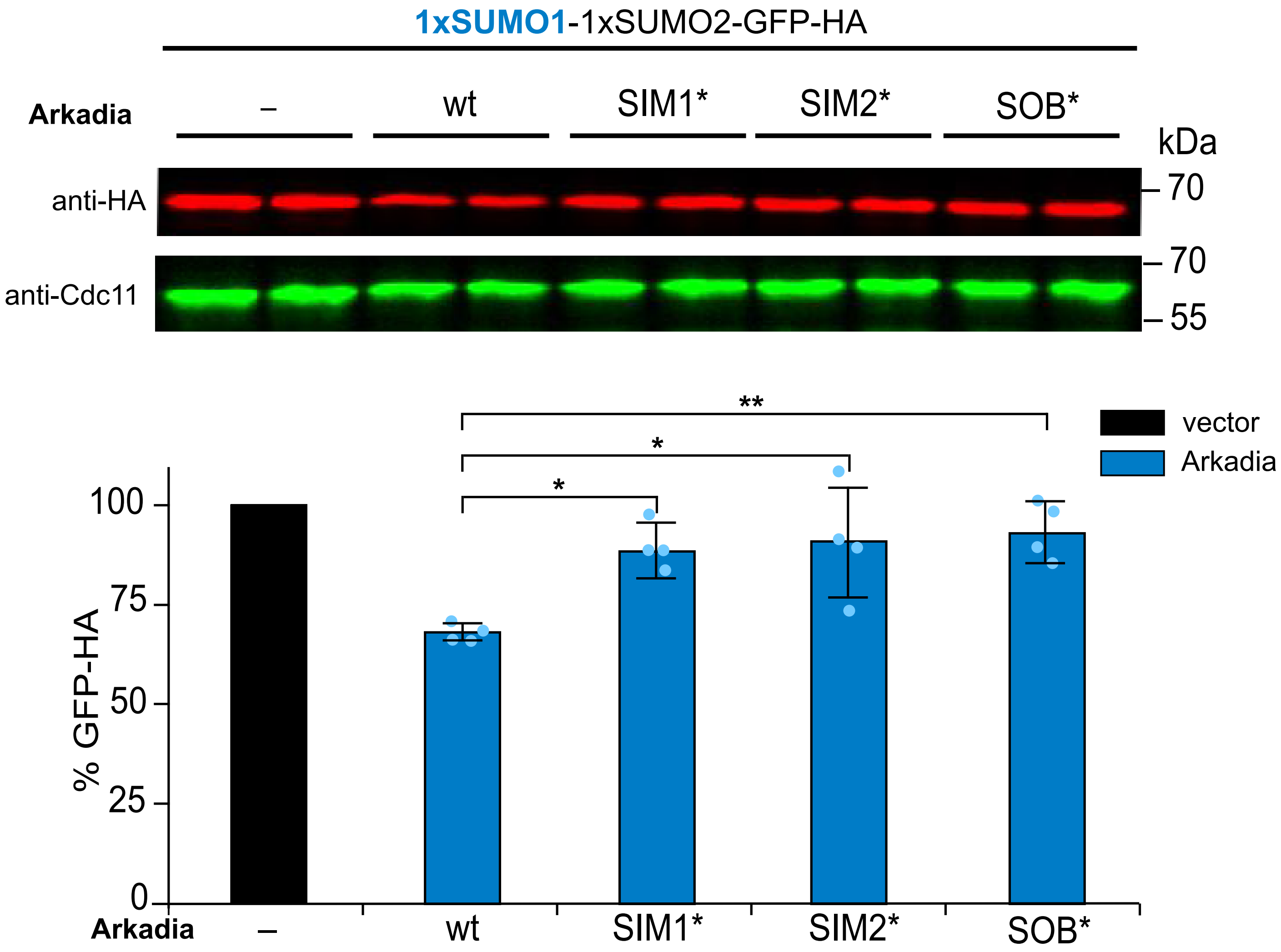

Supplementary Fig. 7

Arkadia's SIM1, SIM2 and SOB motifs all contribute to the targeting of a substrate tagged with a dimeric SUMO1-SUMO2 chain. Shown is comparative analysis of targeting efficiencies of GFP-HA substrate carrying a dimeric SUMO1-SUMO2 chain by wild-type Arkadia, its SOB (SOB*), or SIM (SIM1* or SIM2*) mutant derivatives upon their co-expression in yeast cells. SOB* and SIM* mutations were the same as shown in Fig. 2 and Fig. 3, respectively. Levels of the SUMO1-SUMO2-GFP-HA substrate were determined by quantitative anti-HA Western blotting as described in the legend to Figure 1c. Bars represent the means of data obtained with independent transformants (n=4). Standard deviations (SD) are indicated by error bars. An asterisk indicates significant differences with p-values < 0.1, two asteriks differences with p-values < 0.01. Statistical significance was determined with unpaired and two-sided Student's t-tests. Source data are provided as a Source Data file.

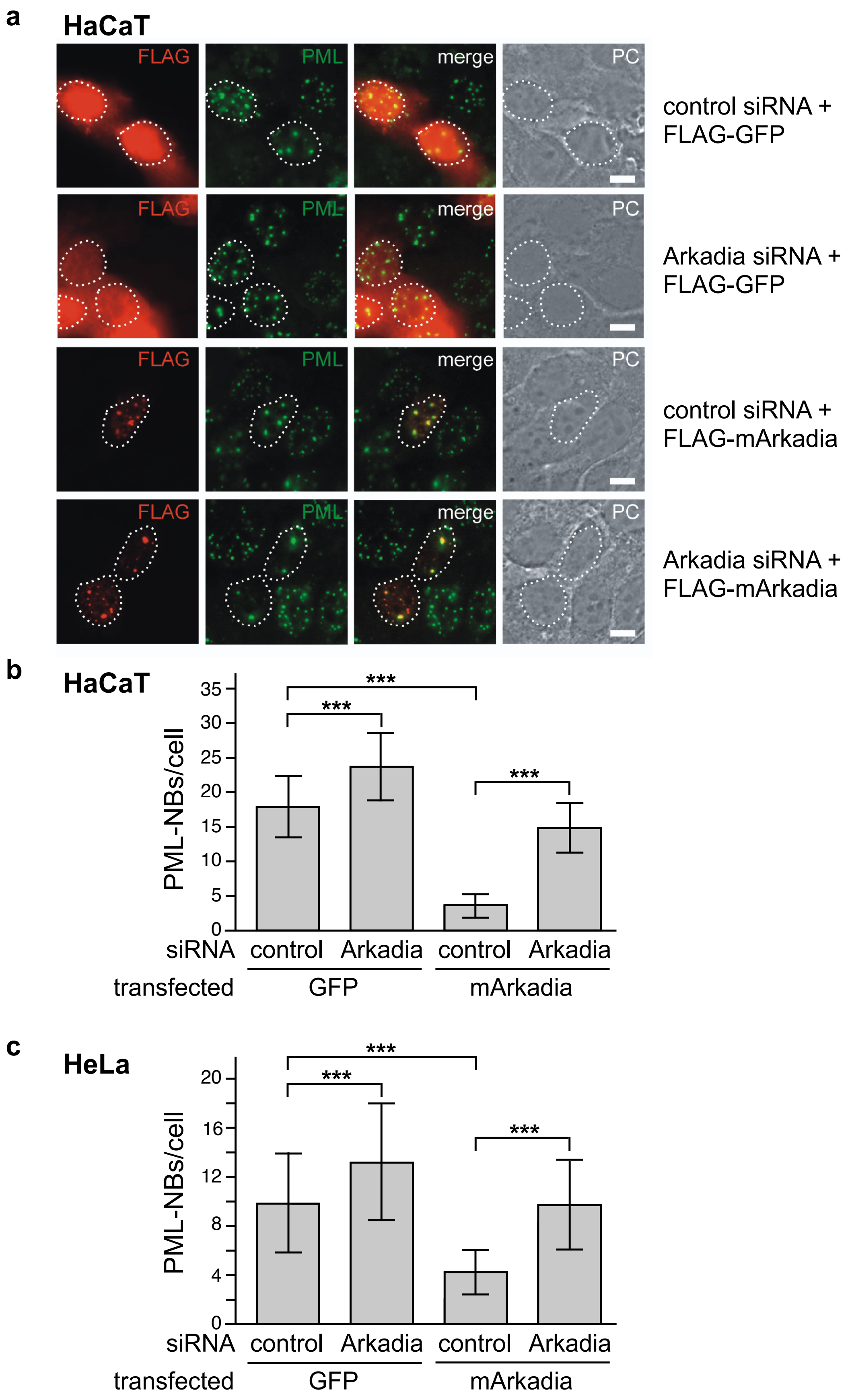

Supplementary Fig. 8. Knockdown experiments show that endogenous Arkadia contributes to the regulation of PML nuclear bodies. (Legend on the following page).

Supplementary Fig. 8

Knockdown experiments show that endogenous Arkadia contributes to the regulation of PML nuclear bodies. a HaCaT cells were co-transfected with plasmids expressing either FLAG-GFP, wild-type (wt) mouse FLAG-Arkadia and either a control siRNA or an siRNA directed against endogenous human Arkadia. Cells were fixed and co-stained using mouse anti-FLAG and rabbit anti-PML antibodies. Nuclei of transfected cells positive for FLAG staining are highlighted by dashed lines. Scale bar, 10 µm. **b** Quantitative analyses of the number of PML-NBs in at least 20 cells from each of the samples shown in **a** (upper part), or **c** from at least 55 cells each of identically treated samples of HeLa cells (no images shown). Bars represent the means. Standard deviations (SD) are indicated by error bars. Three asterisks indicate significant differences with p-values < 0.001 Statistical significance was determined with unpaired and two-sided Student's t-tests. Source data for **b** and **c** are provided as a Source Data file.

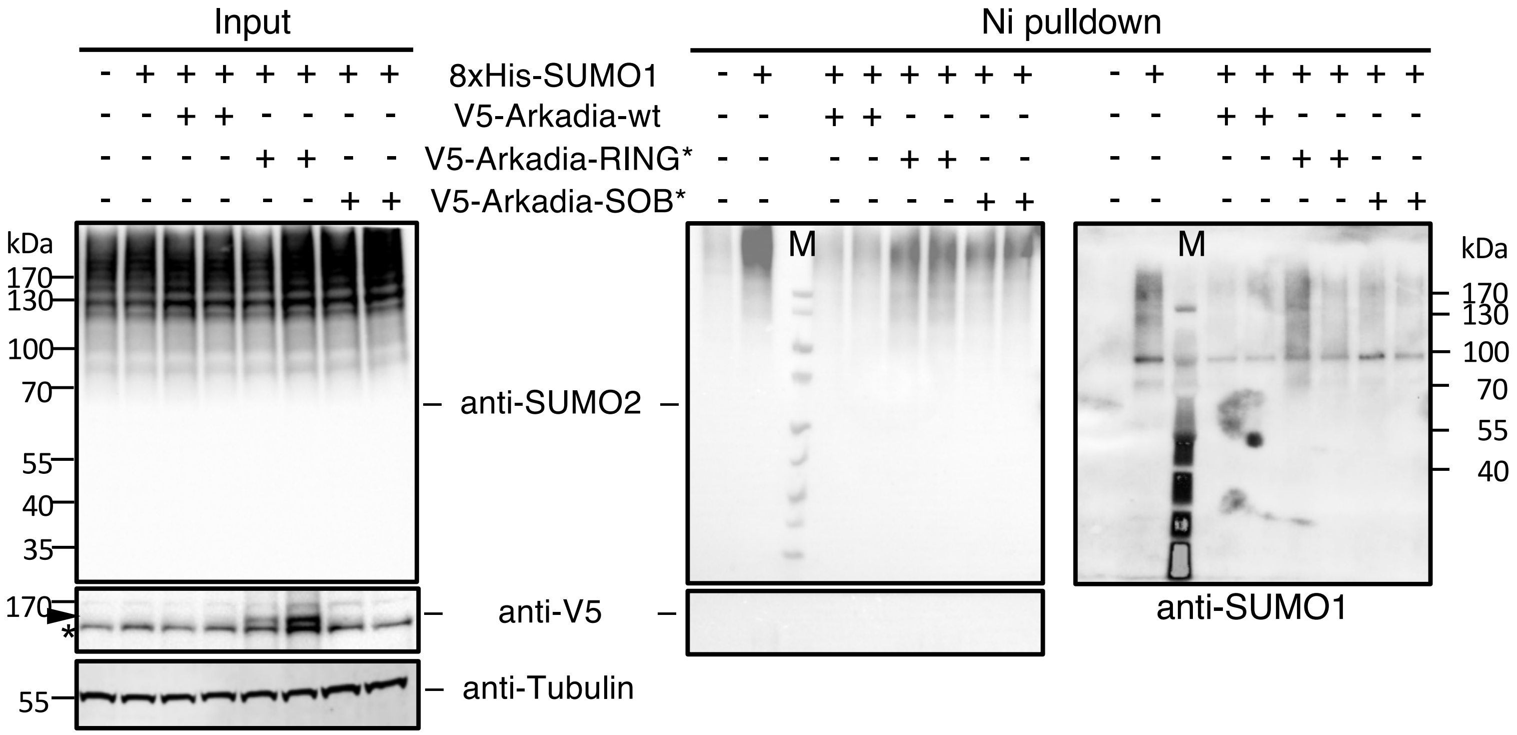

Supplementary Fig. 9

Expression of Arkadia reduces the levels of SUMO1-capped SUMO2 conjugates in human 293T cells in a RING- and SOB-dependent manner. Stable transfectants expressing 8xHis-SUMO1 and as a cleavable linear fusion with either V5-Arkadia (wt), or its RING (RING*) or SOB (SOB*) mutants, or no Arkadia (samples each), were lysed under denaturing conditions (8 M urea). Extracts samples (Input) were analysed with anti-SUMO2, anti-V5, and anti-tubulin antibodies (left panels). An arrowhead marks the position of V5-Arkadia-RING*, which is detected in higher amounts than its wild-type or SOB* counterparts indicating that this ligases mediates its own downregulation. Extracts were subjected to Ni-NTA pulldown. Eluted proteins were analyzed with SUMO2, SUMO1, and V5 antibodies (right panels). Note that expression as a cleavable fusion to Arkadia reduces the expression level of 8xHis-SUMO1 as compared to a cell line just expressing mature 8His-SUMO1 likely due to differences in mRNA stability or translation. M, size marker. Source data are provided as a Source Data file.

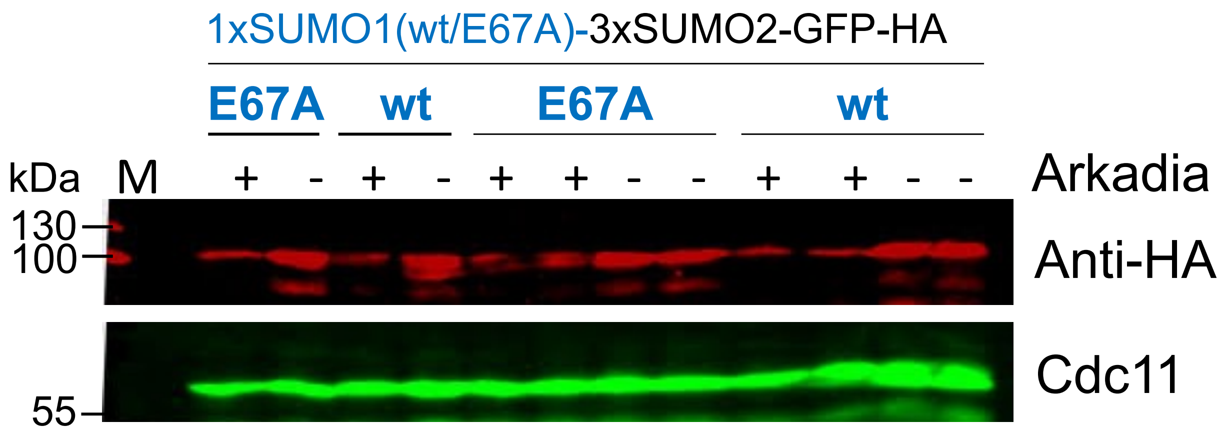

Supplementary Fig. 10

SUMO1-E67A mutation does not interfere with recognition by Arkadia. Analysis of levels of SUMO1-3xSUMO2-GFP substrate either with wild-type SUMO1 (wt) or its E67A mutant version in wild-type yeast cells transformed either with empty vector (-) or a plasmid expressing Arkadia (+). Proteins from four independent transformants were analysed by western blotting with anti-HA and Cdc11 antibodies (loading control). Source data are provided as a Source Data file.

Supplementary Table 1: Plasmids used in this study

Plasmid Name	Description	vector
pAS12	P_{CUP1}-*FLAG-6xHis-1xSUMO1-GFP-2xHA*-T_{CYC1}	YCplac22 [2] (*CEN/TRP1*)
pAS17	P_{CUP1}-*FLAG-6xHis-2xSUMO2-GFP-2xHA*-T_{CYC1}	YCplac22
pAS18	P_{CUP1}-*FLAG-6xHis-3xSUMO2-GFP-2xHA*-T_{CYC1}	YCplac22
pAS19	P_{CUP1}-*FLAG-6xHis-4xSUMO2-GFP-2xHA*-T_{CYC1}	YCplac22
pAS20	P_{CUP1}-*FLAG-6xHis-1xSUMO1-1xSUMO2-GFP-2xHA*-T_{CYC1}	YCplac22
pAS21	P_{CUP1}-*FLAG-6xHis-1xSUMO1-2xSUMO2-GFP-2xHA*-T_{CYC1}	YCplac22
pAS22	P_{CUP1}-*FLAG-6xHis-1xSUMO1-3xSUMO2-GFP-2xHA*-T_{CYC1}	YCplac22
pAS69	P_{CUP1}-*3xSUMO2-GFP-2xHA*- T_{CYC1}	YCplac22
pAS73	P_{CUP1}-*1xSUMO1-3xSUMO2-GFP-2xHA*-T_{CYC1}	YCplac22
pAS137	P_{CUP1}-*1xSUMO2(D71H)- 2xSUMO2-GFP-2xHA*-T_{CYC1}	YCplac22
pAS141	P_{CUP1}-*1xSUMO1(H75D)- 3xSUMO2-GFP-2xHA*-T_{CYC1}	YCplac22
pKG87 [3]	P_{CUP1}-*RNF4*-T_{CYC1}	YEplac181 [2] (2μ/*LEU2*)
pAS23	P_{CUP1}-*Arkadia*-T_{CYC1}	YEplac181
pAS40	P_{CUP1}-*Arkadia(SIM1*)*-T_{CYC1}	YEplac181
pAS41	P_{CUP1}-*Arkadia(SIM2*)*-T_{CYC1}	YEplac181
pAS153	P_{CUP1}-*Arkadia(SOB*)*-T_{CYC1}	YEplac181
pAS160	P_{CUP1}-*4xSUMO1-2xHA*-T_{CYC1}	YCplac22
pAS162	P_{CUP1}-*1xSUMO2-3xSUMO1-GFP-2xHA*-T_{CYC1}	YCplac22
pJD662	P_{CUP1}-*FLAG-6xHis-1xSUMO2-* $Pent_{COMP}$-*GFP-2xHA*-T_{CYC1}	YCplac22
pJD664	P_{CUP1}-*FLAG-6xHis-1xSUMO1-3xSUMO2*-$Pent_{COMP}$-*GFP-2xHA*-T_{CYC1}	YCplac22
pJD665	P_{CUP1}-*1xSUMO1*-$Pent_{COMP}$-*GFP-2xHA*-T_{CYC1}	YCplac33 [2] (*CEN/URA3*)
pJD667	P_{CUP1}-*RNF4-GFP*-T_{CYC1}	YEplac181

pJD668	*P_{CUP1}-Arkadia-GFP-T_{CYC1}*	YEplac181
pGEX-TN-mArkadia(285-416)	*P_{tac}-GST-TEV-Arkadia(M.m.)(285-416)*	pGEX-4T-2;<br>TEV after of thrombin
pGEX-TN-mArkadia(285-416) -SOB*	*P_{tac} - GST-TEV-TN-Arkadia(M.m.)(285-416) -SOB**	pGEX-4T-2;<br>TEV after of thrombin
pGEX-4T-2-2xSUMO2(ΔN11) [4]	*P_{tac}-GST-2xSUMO2(ΔN11)*	pGEX-4T-2<br>GE Healthcare
pGEX-4T-2-3xSUMO2(ΔN11) [4]	*P_{tac}-GST-3xSUMO2(ΔN11)*	pGEX-4T-2 (Sigma)
pGEX-TN-1xSUMO1(ΔN15)-1xSUMO2(ΔN11)	*P_{tac}-GST-1xSUMO1(ΔN15)-1xSUMO2(ΔN11)*	pGEX-4T-2;<br>TEV instead of thrombin
pGEX-TN-1xSUMO1(ΔN15)-2xSUMO2(ΔN11)	*P_{tac}-GST-1xSUMO1(ΔN15)-2xSUMO2(ΔN11)*	pGEX-4T-2;<br>TEV instead of thrombin
pEGFP-C1-FLAG	*P_{CMV}-EGFP-FLAG*	pEGFP-C1 (Clontech)
pCDEF3-FLAG-mRNF111 [5] provided by Masao Saitoh	*P_{CMV}-FLAG-Arkadia (M.m.),*	*pCDNA3*
pCDNA-HA-hRNF111	*pCDNA4-HA-hRNF111 (wt)*	*pCDNA4/TO* (Thermo Fisher Scientific)
pCDNA-HA-hRNF111	*pCDNA4-HA-hRNF111-SOB**	*pCDNA4/TO*
pcDNA5-8His-SUMO1	*pcDNA5/FRT/TO-8His-SUMO-P2AV5*	pcDNA5/FRT/TO (Thermo Fisher Scientific)
pcDNA5-8His-SUMO1-V5-Arkadia-wt	*pcDNA5/FRT/TO-8His-SUMO-P2AV5-hArkadia wt*	pcDNA5/FRT/TO
pcDNA5-8His-SUMO1-V5-Arkadia-RING*	*pcDNA5/FRT/TO-8His-SUMO-P2AV5-hArkadia RING**	pcDNA5/FRT/TO
pcDNA5-8His-SUMO1-V5-Arkadia-SOB*	*pcDNA5/FRT/TO-8His-SUMO-P2AV5-hArkadia SOB**	pcDNA5/FRT/TO

Supplementary Table 2: Oligonucleotides used in this study

Name	Sequence 5’ – 3’	Description
AS3856	AACTGCAGATGAGTAAAGGAGAAGAACTTTTCAC	FP PstI GFP
JD1942	CGCGGTACCTTTGTATAGTTCATCCATGCCATG	RP KpnI GFP
JD168	CGCGGTACCTGGGAATGAGTAAAGGAGAAGAAC	FP KpnI GFP
JD167	CGCTCTAGATTTATTTGTATAGTTCATC	RP XbaI GFP
JD4762	CGCGGATCCATGACAAGAaAGCGTCGTGGTGGAGC	FP BamHI RNF4
JD4763	CGCGGTACCCTATATAAATGGGGTGATACCGTTTGTGGTTGATCTTTTTCC	RP KpnI RNF4
AS3748	CGCGGTACCCACTTTCACTTGGCAGCTGGGCCTC	RP KpnI hArkadia
AS3747	GCCAGTGGACCTGAGCAACAGTGG	FP Arkadia (internal)
JD3814	GAAGACACAGAGGAAAAATGTACGATATCTTTGTCTATTTTAGAGGAAGG	FP hArkadia (RING*)
JD3815	CCTTCCTCTAAAATAGACAAAGATATCGTACATTTTTCCTCTGTGTCTTC	RP hArkadia (RING*)
JD3857	GCGGGATCCATGCATCACCATCACCATCACGCCATGGCCGACGAAAAGCCCAAGGAAGG	FP BamHI 6xHis-SUMO2
JD3858	CGCGCTGCAGTCCCGTCTGCTGTTGGAACACATCAA	RP PstI SUMO2
JD3859	CGCGATGCATACTGAGAACAACGATCATATTAATTTG	FP NsiI SUMO2
JD3860	GCGGGATCCATGCATCACCATCACCATCACGCCATGTCTGACCAGGAGGCAAAACCTTC	FP BamHI 6xHis-SUMO1
JD3861	CGCGCTGCAGCCCCGTTTGTTCCTGATAAACTTCAA	RP SUMO1 PstI
AS3934	GAAGTATTGATGAAGCGGCCGCGGTGATAGAAGCTTCCTCCACTCCCC	FP hArkadia (SIM1*)
AS3935	GCTTCTATCACCGCGGCCGCTTCATCAATACTTCCTGAGGGAACAGCTCC	RP hArkadia (SIM1*)
AS3936	GCAGCAGAAGTTGCCGCGGCGACCGTTGATGAAGATGAACCTACTGTAG	FP hArkadia (SIM2*)
AS3937	CTTCCATCAACGGTCGCCGCGGCAACTTCTGCTGCATTCTGCCTC	RP hArkadia (SIM2*)
AS4411	CAACTGACAGTGAAGCGGAGATTGCAACAGTTGGAGAAAGCTATCGGTCTCG	FP hArkadia (SOB*)
AS4412	CGAGACCGATAGCTTTCTCCAACTGTTGCAATCTCCGCTTCACTGTCAGTTG	RP hArkadia (SOB*)
ML422	GCGGAGCTCCATTACCGACATTTGGGCG	FP SacI P_{CUP1}
AS4009	CGGGATCCAGAATTCGTTACAGTTTGTTTTTCTTAA	RP BamHI P_{CUP1}
AS4030	CGGGATCCATGTCTGACCAGGAGGCAAAACCTTCAACTGAGG	FP BamHI SUMO1
AS4031	CGGGATCCATGGCCGACGAAAAGCCCAAGGAAGGAGTC	FP BamHI-SUMO2
AS4218	GGGCAACCAATCAATGAAACAC ACACACCTGCACAGTTGGAAATGG	FP (D71H) SUMO2
AS4219	CCATTTCCAACTGTGCAGGTGTGTGTGTTTCATTGATTGGTTGCCC	RP (D71H) SUMO2
AS4220	TCACTCAGGTTTCTCTTTGCGGGTCAGAGAATTGCTGATAA	FP (E67A) SUMO1
AS4221	TTATCAGCAATTCTCTGACCCGCAAAGAGAAACCTGAGTGA	RP (E67A) SUMO1
AS4325	CAGAGAATTGCTGATAATGATACTCCAAAAGAACTGGG	FP (H75D) SUMO1
AS4326	CCCAGTTCTTTTGGAGTATCATTATCAGCAATTCTCTG	RP (H75D) SUMO1
JD4741	CGCGGTACCAGGCCATGGATTTAGCTCCACAAATGCTTCGAGAACTCCAGGAGACTA	FP KpnI-NcoI COMP
JD4742	CGCGGTACCCGCAAGCGTCACATTCCATCACC	RP KpnI COMP
GP0746	AAGGAAAAAAGCGGCCGCATGTCTCAATGGACTCCTGAATATAAGGAGCTCTACACC	FP NotI hArkadia
GP0747	CCGCTCGAGTCAACTTTCACTTGGCAGCTGGGCCTCAATGTC	RP XhoI hArkadia
GP0629	CGGGATCCAAGAAGGAAGGTGAATATATTAAACTCAAAGTCATTGGAC	FP BamHI ΔN15-SUMO1
GP0630	GAAGATCTACCCCCCGTTTGTTCCTGATAAACTTCAATCACATC	RP BglII SUMO1
GP0625	CGGGATCCACTGAGAACAACGATCATATTAATTTGAAGGTGGCG	FP BamHI ΔN11-SUMO2
GP0626	GAAGATCTACCTCCCGTCTGCTGTTGGAACACATC	RP BglII SUMO2
GP0921	CGGGATCCAGCAACCCCGCTGCTCCC	FP BamHI mArkadia (int.)
GP0922	GGAATTCGGTAGATGGATTTGAATTGTTAATGGAAGC	RP EcoRI mArkadia (int.)
GP1001	CGGGATCCATGTCTCAATGGACTCCTGAATTTAACGAGC	FP BamHI mArkadia
GP1007	CCTCAACTGACAGTGAAGCGGAGATTGCTACAGTTGGAGAAAGC	FP mArkadia SOB*
GP1008	GCTTTCTCCAACTGTAGCAATCTCCGCTTCACTGTCAGTTGAGG	RP mArkadia SOB*
JD4846	CGCGCTTAAGACTAAGAGGTGGTATGCATCACCATCACCATCACCATC ACGCCATGTC	FP 8His-SUMO1
NG2102	TTTTCTCGAGATGTCTCAATGGACTCCTGAATATAACGAGCTCTACACCTTAAAAGTGGA	FP XhoI hArkadia
NG2103	TTTTTTTGCGGCCGCTCAACTTTCACTTGGCAGCTGGGCCT	RP NotI hArkadia
NG2104	TTTTTTGCTAGCACCATGCATCACCATCACCAC	FP NheI 8His
NG2105	TTTTCTCGAGGGTGCTGTCCAGGCCCAGCAGTGGGTTAGGGATGGGCTTGCCTGCACCCCC CGTTTGTTC	RP XhoI SUMO1-V5

Supplementary References

[1] Ramos, P.C., Höckendorff, J., Johnson, E., Varshavsky, A., & Dohmen, R.J. Ump1p is required for maturation of the 20S proteasome, and becomes its substrate upon completion of the assembly. Cell 20, 489-499 (1998).

[2] Gietz, R.D. & Sugino, A. New yeast-*Escherichia coli* shuttle vectors constructed with in vitro mutagenized yeast genes lacking six-base pair restriction sites. *Gene* 74, 527-534 (1988).

[3] Weisshaar, S.R., Keusekotten, K., Krause, A., Horst, C., Springer, H.M., Göttsche, K., Dohmen, R.J., and Praefcke, G.J. Arsenic trioxide stimulates SUMO-2/3 modification leading to RNF4-dependent proteolytic targeting of PML. FEBS Lett 582, 3174-3178.

[4] Keusekotten, K., Bade, V.N., Meyer-Teschendorf, K., Sriramachandran, A.M., Fischer-Schrader, K., Krause, A., Horst, C., Schwarz, G., Hofmann, K., Dohmen, R.J., & Praefcke, G.J.K. Multivalent interactions of the SUMO-interaction motifs in RING finger protein 4 determine the specificity for chains of the SUMO. *Biochem J 457*, 207-214 (2014).

[5] Koinuma, D., Shinozaki, M., Komuro, A., Goto, K., Saitoh, M., Hanyu, A., Ebina, M., Nukiwa, T., Miyazawa, K., Imamura, T., & Miyazono, K. Arkadia amplifies TGF-β superfamily signaling through degradation of Smad7. *EMBO J* 22, 6458-6470 (2003).
