# Supplementary material for: Arkadia/RNF111 is a SUMO-targeted ubiquitin ligase with preference for substrates marked with SUMO1-capped SUMO2/3 chain
Source: Nat Commun. 2019 Aug 15;10:3678. doi: 10.1038/s41467-019-11549-3 (PMC6695498; doi:10.1038/s41467-019-11549-3)
Supplement: Supplementary file 2 — Reporting summary [file 41467_2019_11549_MOESM2_ESM.pdf]

nature research

Corresponding author(s): R. Jürgen Dohmen, Gerrit J.K. Praefcke, Kay Hofmann

Last updated by author(s): Jul 15, 2019

Reporting Summary

Nature Research wishes to improve the reproducibility of the work that we publish. This form provides structure for consistency and transparency in reporting. For further information on Nature Research policies, see Authors & Referees and the Editorial Policy Checklist.

Statistics

For all statistical analyses, confirm that the following items are present in the figure legend, table legend, main text, or Methods section.

n/a	Confirmed	
☐	☒	The exact sample size (n) for each experimental group/condition, given as a discrete number and unit of measurement
☐	☒	A statement on whether measurements were taken from distinct samples or whether the same sample was measured repeatedly
☐	☒	The statistical test(s) used AND whether they are one- or two-sided<br>*Only common tests should be described solely by name; describe more complex techniques in the Methods section.*
☒	☐	A description of all covariates tested
☒	☐	A description of any assumptions or corrections, such as tests of normality and adjustment for multiple comparisons
☐	☒	A full description of the statistical parameters including central tendency (e.g. means) or other basic estimates (e.g. regression coefficient) AND variation (e.g. standard deviation) or associated estimates of uncertainty (e.g. confidence intervals)
☒	☐	For null hypothesis testing, the test statistic (e.g. F, t, r) with confidence intervals, effect sizes, degrees of freedom and P value noted<br>*Give P values as exact values whenever suitable.*
☒	☐	For Bayesian analysis, information on the choice of priors and Markov chain Monte Carlo settings
☒	☐	For hierarchical and complex designs, identification of the appropriate level for tests and full reporting of outcomes
☒	☐	Estimates of effect sizes (e.g. Cohen's d, Pearson's r), indicating how they were calculated

Our web collection on statistics for biologists contains articles on many of the points above.

Software and code

Policy information about availability of computer code

Data collection	Licor, Zeiss, Phosphoimager
Data analysis	Excel, Licor, Microscopical images were processed with imageJ, Excel

For manuscripts utilizing custom algorithms or software that are central to the research but not yet described in published literature, software must be made available to editors/reviewers. We strongly encourage code deposition in a community repository (e.g. GitHub). See the Nature Research guidelines for submitting code & software for further information.

Data

Policy information about availability of data

All manuscripts must include a data availability statement. This statement should provide the following information, where applicable:

- Accession codes, unique identifiers, or web links for publicly available datasets
- A list of figures that have associated raw data
- A description of any restrictions on data availability

The authors declare that the data supporting the findings of this study are available within the article and Supplementary Information Files.

Field-specific reporting

Please select the one below that is the best fit for your research. If you are not sure, read the appropriate sections before making your selection.

☒ Life sciences ☐ Behavioural & social sciences ☐ Ecological, evolutionary & environmental sciences

Life sciences study design

All studies must disclose on these points even when the disclosure is negative.

Sample size	Sample sizes and number of replicates were chosen on the basis that reproducibility can be established and statistical significance can be reached.
Data exclusions	In the quantitative western blot analyses, transformants not expressing the reporter protein were excluded. Also, bands of proteins which were apparently not blotted properly due to blotting inhomogeneity or air bubbles were excluded from the statistical analysis.
Replication	All data in the manuscript have been reproduced at least once; most data were produced from at least three independent biological replicates (as indicated in the figure legends).
Randomization	Samples were not randomized.
Blinding	Cover slips used for PML body counting were blinded by another researcher to ensure unbiased counting.

Reporting for specific materials, systems and methods

We require information from authors about some types of materials, experimental systems and methods used in many studies. Here, indicate whether each material, system or method listed is relevant to your study. If you are not sure if a list item applies to your research, read the appropriate section before selecting a response.

Materials & experimental systems

n/a	Involved in the study	
☐	☒	Antibodies
☐	☒	Eukaryotic cell lines
☒	☐	Palaeontology
☒	☐	Animals and other organisms
☒	☐	Human research participants
☒	☐	Clinical data

Methods

n/a	Involved in the study	
☒	☐	ChIP-seq
☒	☐	Flow cytometry
☒	☐	MRI-based neuroimaging

Antibodies

Antibodies used	mouse anti-HA (16B12); rat anti-HA (3F10); mouse anti-V5, mouse Anti-SUMO1 and anti-SUMO2 were produced with hybridoma cell lines 21C7 and 8A2from Developmental Studies Hybridoma Bank (antibody registry ID: AB_2198257 and AB_2198421, respectively); mouse anti-Ubiquitin (P4D1) Santa Cruz Biotechnology, sc-8017; rabbit anti-PML (PML Antibody AbVantage™ Pack, A310-390A, Bethyl Laboratories Inc), mouse anti-FLAG (M2, Sigma–Aldrich) primary antibodies. Goat anti-Mouse IgG, Cross-Adsorbed Secondary Antibody, Alexa Fluor 546 ThermoFisher Scientific Catalog # A-11003; Donkey anti-Rabbit IgG, Cross-Adsorbed Secondary Antibody, Alexa Fluor 647 Catalog # A-31571. Cdc11, Tubulin, anti-mouse IGG...; anti-rabbit IGG
Validation	Ubiquitin, P4D1: https://datasheets.scbt.com/sc-8017.pdf; PML: A310-390A, https://www.bethyl.com/product/pdf/A310-390A.pdf, Mathieu et al. Cell Death and Disease (2014) 5, e1061; doi:10.1038/cddis.2014.29

Eukaryotic cell lines

Policy information about cell lines

Cell line source(s)	HeLa B cells: European Collection of Authenticated Cell Cultures (ECACC) Catalogue No.: 85060701, HaCat, Flp-InTM T-RExTM 293 cell line (Thermo Fisher Scientific Cat. R789007).<br>Yeast strains were generated in our laboratory.
Authentication	None of the human cell lines used were authenticated in our laboratories. Yeast strains were verified by PCR analyses.
Mycoplasma contamination	Human cell lines were routinely tested for Mycoplasma contamination using a Mycoplasma PCR kit from AppliChem.
Commonly misidentified lines (See ICLAC register)	No commonly misidentified cell lines were used.
